# Temporal Trends and Disparities in Mortality Associated With Type 2 Diabetes and Ischemic Heart Disease in the United States (1999−2024)

**DOI:** 10.1002/clc.70513

**Published:** 2026-11-05

**Authors:** Muhammad Shaheer Bin Faheem, Syed Tawassul Hassan, Syed Atta Ur Rafe, Sivaram Neppala, Nafila Zeeshan, Akshay Kumar, Nimra Shafi, Sumaya Samadi, Yasar Sattar

**Affiliations:** ^1^ Karachi Institute of Medical Sciences, KIMS Karachi Sindh Pakistan; ^2^ Karachi Medical and Dental College Karachi Metropolitan University Karachi Sindh Pakistan; ^3^ Jinnah Sindh Medical University, JSMU Karachi Sindh Pakistan; ^4^ Division of Cardiology The University of Texas Health Sciences Center San Antonio Texas USA; ^5^ Dow University of Health Sciences, DUHS Karachi Sindh Pakistan; ^6^ Department of Cardiothoracic Surgery NYU Langone Health New York New York USA; ^7^ Arnot Ogden Medical Center Elmira New York USA; ^8^ Kabul University of Medical Sciences “Abu Ali Ibn Sina” Kabul Afghanistan; ^9^ Department of Cardiology West Virginia University Morgantown West Virginia USA

**Keywords:** cardiology, diabetes, ischemic heart disease, T2DM

## Abstract

**Introduction:**

Patients with co‐existing type 2 diabetes mellitus (T2DM) and ischemic heart disease (IHD) face a higher mortality risk than those with either condition alone. We examined US mortality trends and disparities in adults with both conditions, 1999–2024.

**Methods:**

CDC WONDER death certificate data were used to identify adults aged ≥ 45 years with both T2DM (ICD‐10 E11) and IHD (I20–I25) listed as underlying or contributing causes. Age‐adjusted mortality rates (AAMRs) per 100 000 were calculated, and trends were assessed with Joinpoint regression (permutation test) to estimate annual percent change (APC) and average annual percent change (AAPC).

**Results:**

T2DM and IHD were co‐listed on 828 250 death certificates. The AAMR rose from 18.87 in 1999 to 37.65 in 2021 and was 32.97 in 2024 (AAPC 2.06%; 95% CI, 1.68–2.61). AAMRs increased until 2005 (APC 3.51%), declined until 2015 (APC −0.93%), surged until 2021 (APC 9.06%), and declined thereafter (APC −4.04%). AAMRs were higher in males than females (36.33 vs. 18.53) and highest among non‐Hispanic American Indian or Alaska Native adults (44.27). Age‐specific rates rose from 2.74 (45–54 years) to 149.59 (≥ 85 years). AAMRs were higher in non‐metropolitan than metropolitan areas (30.89 vs. 22.59; 1999–2020) and highest in the West (31.24) and Midwest (30.57).

**Conclusions:**

AAMRs increased overall from 1999 to 2024 despite a decline after 2021, with marked demographic and geographic disparities. Targeted interventions and equitable access to cardioprotective therapies are needed for high‐risk populations.

## Graphical abstract

Among US adults aged ≥ 45 years, mortality with co‐existing type 2 diabetes and ischemic heart disease rose from 1999 to 2024, peaking in 2021. Rates were highest among men, American Indian or Alaska Native adults, non‐metropolitan residents, and the West and Midwest, underscoring the need for equitable cardioprotective care.

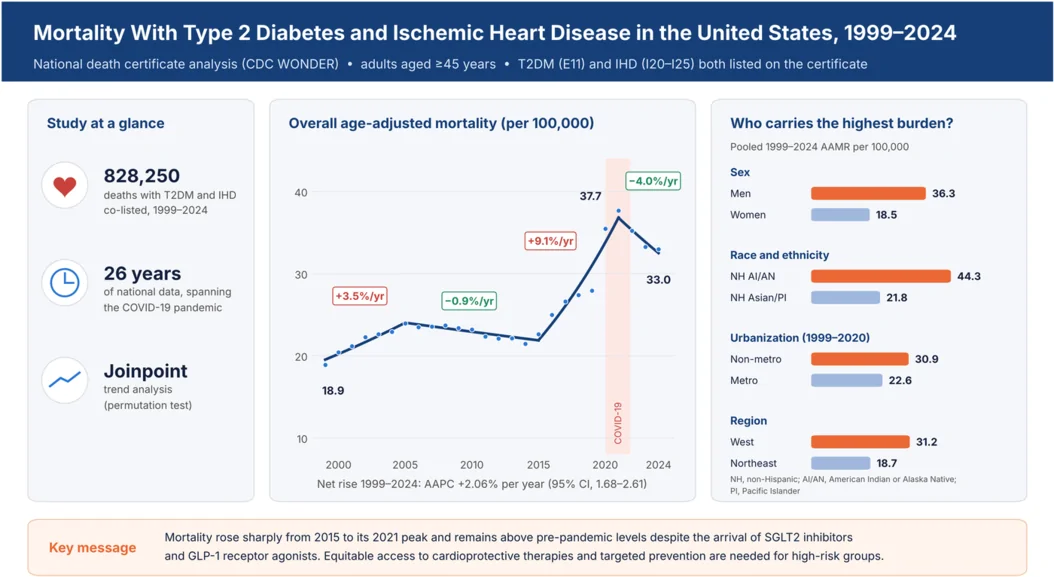

## 1 Introduction

Type 2 diabetes mellitus (T2DM) is one of the leading health problems, currently affecting 38.4 million people in the United States (US), with an estimated 11.6% of the population having the disease in 2021 [1]. T2DM is not just a metabolic disease, but also a major risk factor for atherosclerotic cardiovascular disease, of which ischemic heart disease (IHD) is the leading component and the leading cause of death in the United States [2, 3]. A recent study that used US county‐level mortality data from the CDC examined 9 108 644 deaths from IHD between 1999 and 2020 [3]. Given the well‐known link between diabetes mellitus (DM) and IHD, it is important to understand the impact of T2DM on IHD‐related mortality for the development of appropriate public health strategies.

The relationship between IHD and T2DM is one of the most concerning complications of T2DM. DM promotes the development of atherosclerosis and disrupts the restoration of blood flow to ischemic tissue through angiogenesis and vascular remodeling [4, 5]. Additionally, hyperglycemia in DM patients impairs the signaling pathways of hypoxia‐inducible factor 1 (HIF‐1), reducing pro‐angiogenic effects while promoting oxidative stress, inflammation, and endothelial dysfunction. These mechanisms play a key role in increasing IHD severity and IHD mortality in people with DM [6].

Several recent analyses of the CDC Wide‐ranging Online Data for Epidemiologic Research (CDC WONDER) database have described cardiovascular mortality among adults with diabetes. Zhou et al. reported rising cardiovascular mortality among adults aged ≥ 65 years with T2DM from 1999 to 2023 [7], and other studies have examined IHD with concurrent diabetes and dyslipidemia [8] or acute myocardial infarction (MI) by diabetes type [9]. However, these studies either grouped all cardiovascular causes together, excluded middle‐aged adults, or did not examine T2DM‐associated IHD mortality across urbanization and place of death. Because IHD accounts for a large share of diabetes‐related cardiovascular deaths [10], and because middle‐aged adults have the most life‐years to gain from prevention, an IHD‐specific analysis across adults aged 45 years and older remains warranted. Therefore, we analyzed the CDC WONDER database to examine the temporal trends in mortality among adults with both T2DM and IHD listed on their death certificates from 1999 to 2024, a period that extends from before the introduction of sodium−glucose cotransporter‐2 (SGLT2) inhibitors and glucagon‐like peptide‐1 receptor agonists (GLP‐1RAs) through the COVID‐19 pandemic and its aftermath. Our analysis evaluates trends in age‐adjusted mortality rates (AAMRs) while exploring mortality disparities across sex, age, race and ethnicity, urbanization, census region, and all 50 states and the District of Columbia. Through our findings, we aim to inform policymaking bodies about high‐risk populations so they can initiate targeted interventions with the equitable allocation of healthcare resources.

## 2 Methods

### 2.1 Data Source and Study Population

This retrospective, serial cross‐sectional study assessed T2DM‐ and IHD‐related mortality across all 50 US states and the District of Columbia from 1999 to 2024 using the CDC WONDER Multiple Cause of Death files, which contain death certificate information for US residents categorized by the International Classification of Diseases, Tenth Revision (ICD‐10) [11, 12]. Data for 1999–2020 were obtained from the 1999−2020 final file (bridged‐race categories), and data for 2021–2024 from the 2018–2024 single‐race file; both were accessed in October 2026. As this study relied on publicly available, de‐identified data, institutional review board (IRB) approval was not required. The study followed the Strengthening the Reporting of Observational Studies in Epidemiology (STROBE) guidelines.

### 2.2 Data Extraction

We included deaths of US residents aged ≥ 45 years in which both T2DM (ICD‐10 codes E11.0–E11.9) and IHD (ICD‐10 codes I20–I25) were listed anywhere on the death certificate, either as the underlying cause or as a contributing cause of death. In CDC WONDER, the E11 codes were entered in the first multiple‐cause field and I20–I25 in a second field joined by the “AND” operator, so that only certificates listing both conditions were counted; the underlying cause of death was not restricted. Deaths were stratified by sex; race and ethnicity (non‐Hispanic [NH] White, NH Black or African American, NH American Indian or Alaska Native, NH Asian or Pacific Islander, and Hispanic or Latino); 10‐year age group (45–54, 55–64, 65–74, 75–84, and ≥ 85 years); place of death; US Census region (Northeast, Midwest, South, and West); state; and urbanization according to the 2013 National Center for Health Statistics Urban–Rural Classification Scheme (metropolitan: large central, large fringe, medium, and small metro; non‐metropolitan: micropolitan and noncore) [13]. For 2021–2024, single‐race NH Asian and NH Native Hawaiian or Other Pacific Islander decedents were combined to match the bridged‐race NH Asian or Pacific Islander category. Because CDC WONDER does not release population denominators for urbanization categories in the single‐race file, urbanization‐specific rates were analyzed for 1999–2020, whereas death counts by urbanization are reported for the full study period. CDC WONDER suppresses counts of 1–9 deaths; the few suppressed cells in stratified queries were recovered from unsuppressed totals, so no estimate relied on missing data. All query parameters and saved query URLs are provided in Supporting Information S1: Table 9.

### 2.3 Statistical Analysis

Mortality was expressed as crude mortality rates and AAMRs per 100 000 population. Crude rates were calculated by dividing the number of deaths by the corresponding US population, and AAMRs were calculated by direct standardization to the 2000 US standard population [14]. Because age adjustment is not meaningful within narrow age strata, results by age group are presented as age‐specific mortality rates. Pooled AAMRs for the full study period were calculated by direct standardization from 10‐year age‐specific deaths and populations summed across both CDC WONDER files; this method reproduced the period AAMRs generated by CDC WONDER to within 0.04 per 100 000 for every subgroup. Temporal trends were assessed with the Joinpoint Regression Program (version 5.2.0, National Cancer Institute) [15], fitting log‐linear models with heteroscedastic errors based on the standard errors provided by CDC WONDER. Identical pre‐specified settings were applied to all series: grid search, a maximum of four joinpoints (the recommended maximum for 22–26 observations), and a minimum of two observations between joinpoints and from either end of the series. The number of joinpoints was selected by the permutation test (4499 permutations; overall significance level 0.05 with Bonferroni adjustment) [16], and no joinpoints were placed manually (Supporting Information S1: Table 10). Each series (overall, sex, race and ethnicity, age group, urbanization, and region) was modeled independently. Annual percent change (APCs), average annual percent change (AAPCs) for the full period, and their 95% confidence intervals (CIs) were estimated with the empirical quantile method (5001 resamples). As a sensitivity analysis, all models were re‐fitted with the weighted Bayesian information criterion (WBIC) (Supporting Information S1: Table 11). Trends were described as increasing or decreasing when the APC differed significantly from zero (two‐sided *p* < 0.05) and as stable otherwise.

### 2.4 Eligibility and Case Selection

We restricted our analysis to decedents aged 45 years and older, because counts in younger age groups were small and yielded unreliable rate estimates; decedents with unknown age were excluded. Officially registered deaths were queried from 1999 to 2024 in which both T2DM and IHD were listed as underlying or contributing causes of death. Because the database comprises all filed death certificates, no further exclusion criteria were required or applied.

## 3 Results

From 1999 to 2024, T2DM and IHD were co‐listed on 828 250 death certificates of US adults aged 45 years and older (619 812 in 1999–2020 and 208 438 in 2021–2024). Most decedents were males (58.5%), NH White individuals (76.9%), and adults aged 75–84 years (33.5%). Most deaths occurred in metropolitan areas (77.6%) and the South region (33.8%), reflecting the population size of these areas. By place of death, 38.0% of deaths occurred in medical facilities, 34.8% at the decedent's home (the most common single location), and 20.7% in nursing homes or long‐term care facilities (Supporting Information S1: Figure 1 and Table 1.

### 3.1 Twenty‐Six‐Year Trajectory of Age‐Adjusted Mortality in Co‐Occurring T2DM and IHD

Overall, the AAMR rose from 18.87 in 1999 to a peak of 37.65 in 2021 and was 32.97 in 2024, corresponding to an AAPC of 2.06% (95% CI, 1.68–2.61); the pooled AAMR for 1999–2024 was 26.03 (95% CI, 25.98–26.09). The permutation test selected three joinpoints. AAMRs increased significantly from 1999 to 2005 (APC 3.51%; 95% CI, 1.52–8.78), declined from 2005 to 2015 (APC −0.93%; 95% CI, −4.39 to −0.05), rose steeply from 2015 to 2021 (APC 9.06%; 95% CI, 7.35–12.89), and declined significantly from 2021 to 2024 (APC −4.04%; 95% CI, −7.57 to −0.46) (Figure 1, Supporting Information S1: Tables 2, 3).

**Figure 1:**
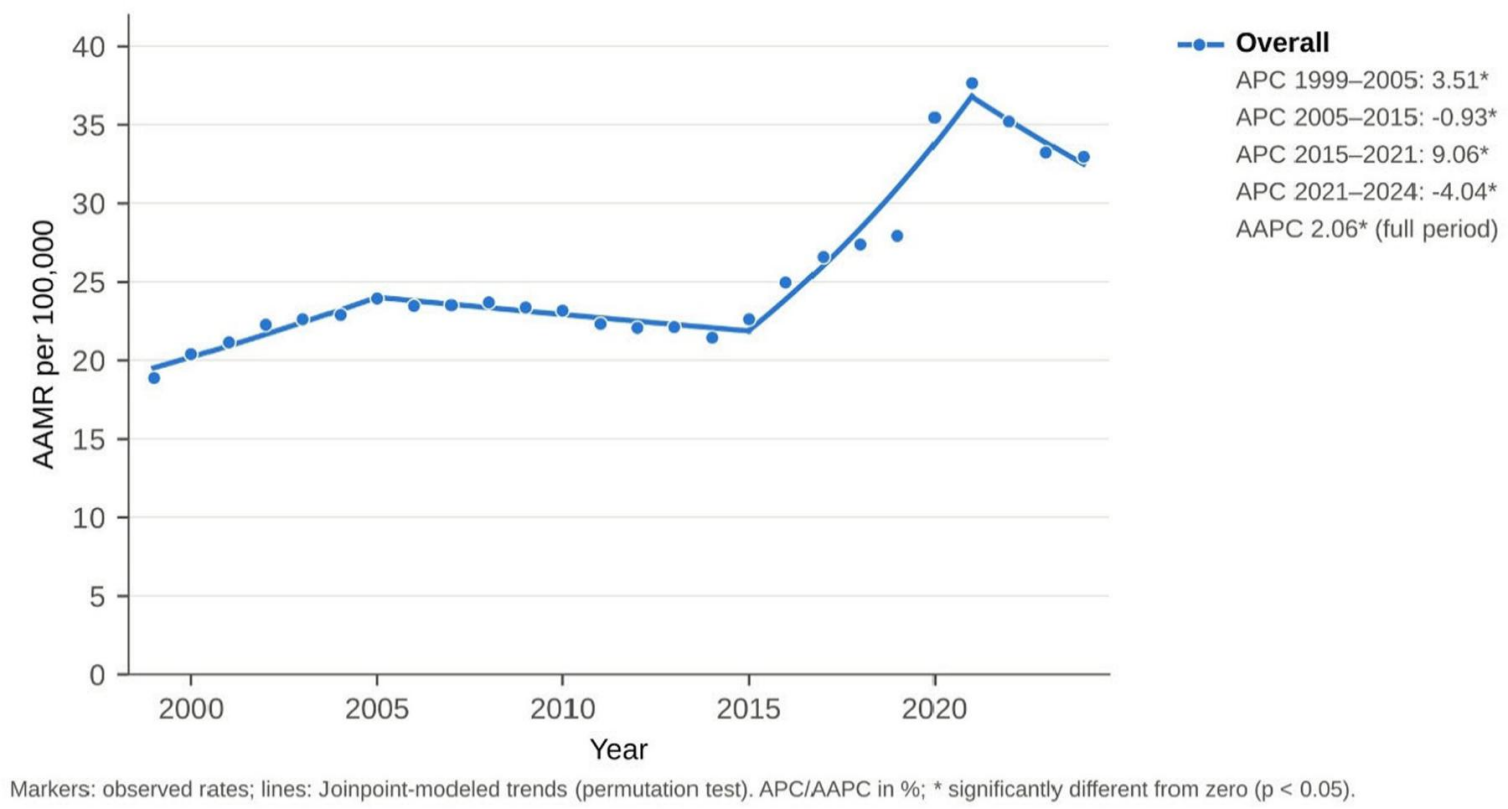
Overall trends in T2DM and IHD‐related age‐adjusted mortality rates per 100 000 among adults aged 45 and above in the United States, 1999 to 2024. Markers show observed rates; lines show Joinpoint‐modeled trends (permutation test). *Indicates that the APC or AAPC is significantly different from zero at *α* = 0.05. AAPC, average annual percent change; APC, annual percent change.

### 3.2 Sex Stratified

The pooled AAMR for males was 36.33 (95% CI, 36.23–36.44), nearly double that of females at 18.53 (95% CI, 18.47–18.59). Among females, AAMRs increased from 1999 to 2005 (APC 2.76%), declined from 2005 to 2015 (APC −2.06%), rose from 2015 to 2021 (APC 7.99%), and declined from 2021 to 2024 (APC −4.38%). Among males, AAMRs increased from 1999 to 2004 (APC 4.92%), remained stable from 2004 to 2015 (APC −0.11%), rose from 2015 to 2021 (APC 9.51%), and declined from 2021 to 2024 (APC −3.84%). The AAPC was higher among males (2.66%) than females (1.13%) (Figure 2, Supporting Information S1: Tables 2, 3).

**Figure 2:**
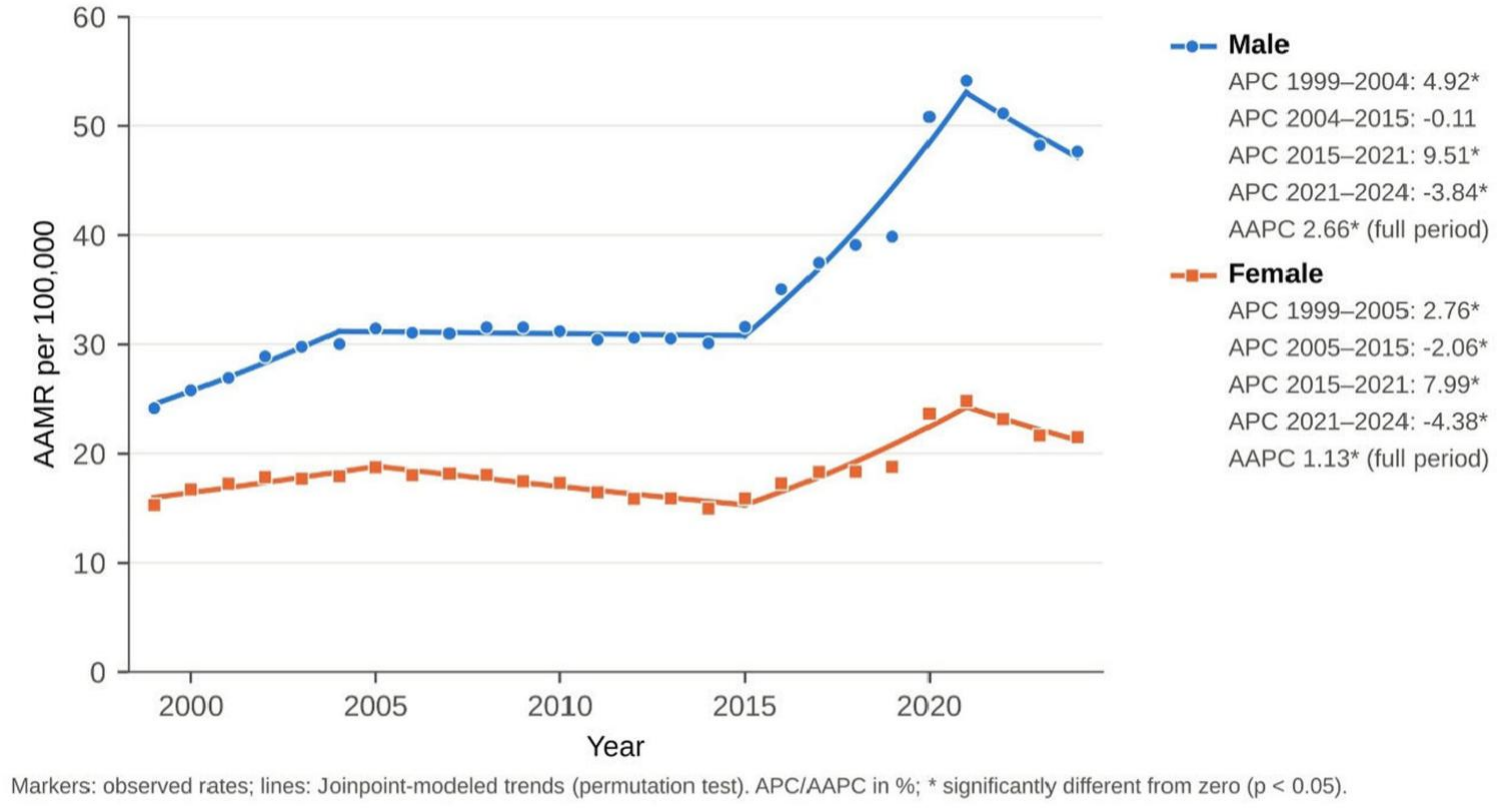
Trends in T2DM and IHD‐related age‐adjusted mortality rates per 100 000, stratified by sex among adults aged 45 and above in the United States, 1999 to 2024. Markers show observed rates; lines show Joinpoint‐modeled trends (permutation test). *Indicates that the APC or AAPC is significantly different from zero at *α* = 0.05. AAPC, average annual percent change; APC, annual percent change.

### 3.3 Race/Ethnicity Stratified

Pooled AAMRs were highest among NH American Indian or Alaska Native adults (44.27; 95% CI, 43.23–45.30), followed by Hispanic (31.26), NH Black or African American (27.34), NH White (25.37), and NH Asian or Pacific Islander adults (21.83). Among NH American Indian or Alaska Native adults, AAMRs rose from 1999 to 2003 (APC 10.31%), remained stable from 2003 to 2014, rose from 2014 to 2021 (APC 7.12%), and declined from 2021 to 2024 (APC −6.78%). NH Black or African American adults showed an increase from 1999 to 2006 (APC 3.76%), a decline from 2006 to 2014 (APC −2.21%), an increase from 2014 to 2021 (APC 7.56%), and a decline from 2021 to 2024 (APC −4.26%).

NH White adults followed a similar pattern, with an increase from 1999 to 2005 (APC 3.53%), a decline from 2005 to 2015 (APC −1.19%), and a sharp rise from 2015 to 2021 (APC 8.98%), followed by a nonsignificant decline from 2021 to 2024 (APC −3.05%). Among NH Asian or Pacific Islander adults, AAMRs increased from 1999 to 2014 (APC 2.79%) and more steeply from 2014 to 2021 (APC 9.77%) before declining from 2021 to 2024 (APC −7.37%). Among Hispanic adults, AAMRs increased from 1999 to 2017 (APC 1.60%), rose steeply from 2017 to 2020 (APC 15.61%), and declined from 2020 to 2024 (APC −4.76%). AAPCs were significant in all groups and highest among NH Asian or Pacific Islander adults (3.40%) (Figure 3, Supporting Information S1: Tables 3, 4).

**Figure 3:**
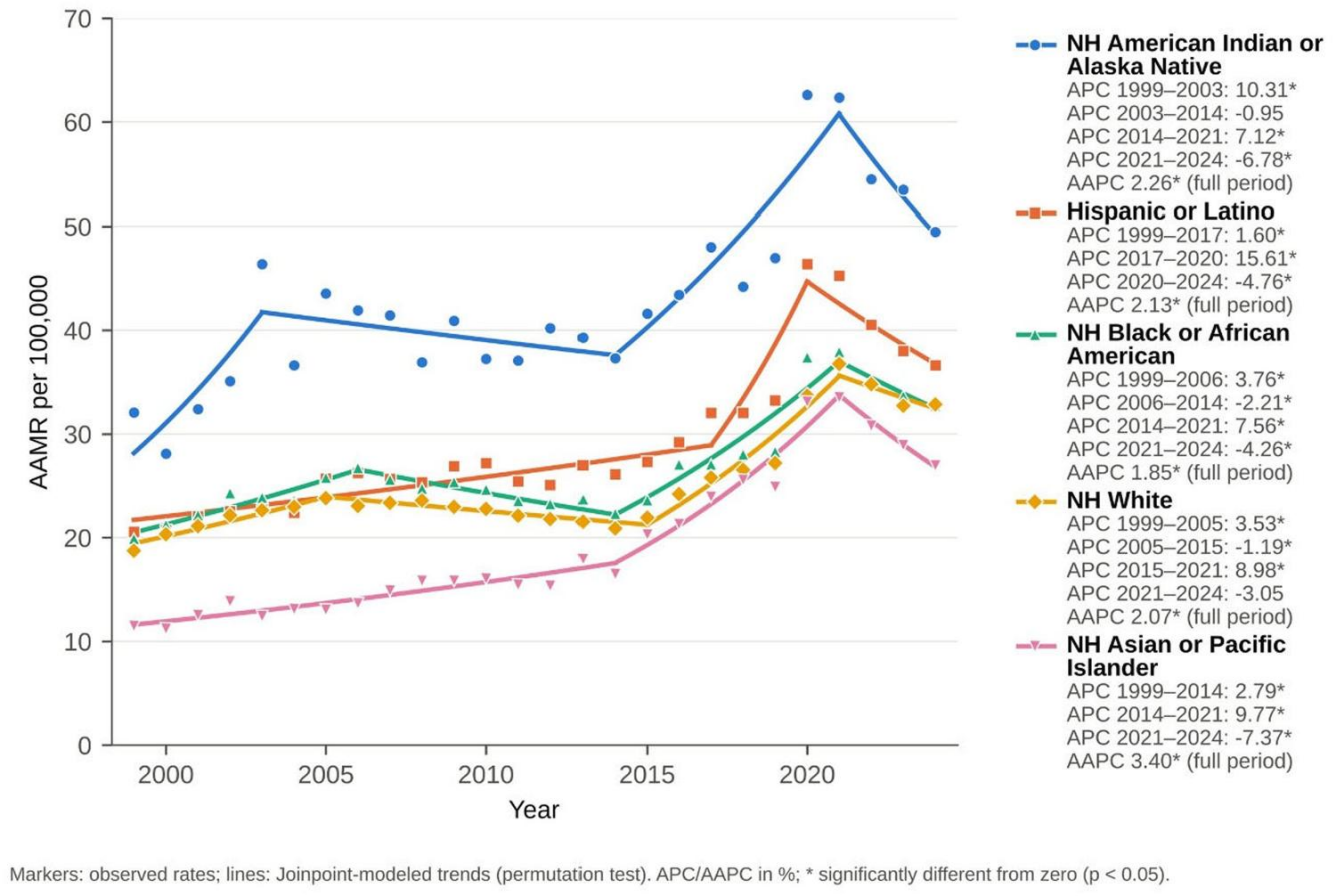
Trends in T2DM and IHD‐related age‐adjusted mortality rates per 100 000, stratified by race and ethnicity among adults aged 45 and above in the United States, 1999 to 2024. Markers show observed rates; lines show Joinpoint‐modeled trends (permutation test). *Indicates that the APC or AAPC is significantly different from zero at *α* = 0.05. AAPC, average annual percent change; APC, annual percent change; NH, non‐Hispanic.

### 3.4 Age Group Stratified

Age‐specific mortality rates rose steeply with age, from 2.74 per 100 000 among adults aged 45–54 years to 10.80, 31.36, 75.12, and 149.59 among those aged 55–64, 65–74, 75–84, and ≥ 85 years, respectively. All age groups followed a similar pattern, with increases in the early 2000s, declining or stable rates until 2014–2015, and steep increases until 2021 (APCs 7.92%–11.87%). The steepest increase from 2015 to 2021 occurred among adults aged 45–54 years (APC 11.87%), who also had the highest AAPC (4.06%). From 2021 to 2024, age‐specific mortality rates declined significantly among adults aged 55–64 (APC −4.07%), 65–74 (APC −5.55%), and 75–84 years (APC −4.50%), whereas the declines among adults aged 45–54 (APC −3.86%) and ≥ 85 years (APC −1.36%) were not significant (Supporting Information S1: Figure 2A,2B, Tables 3, 5).

### 3.5 Geographic Trends

Pooled AAMRs varied more than fourfold across all 50 states and the District of Columbia, from 11.01 in Georgia to 48.58 in West Virginia. States in the top 10th percentile (West Virginia, Iowa, Ohio, Oklahoma, Tennessee, and California) had AAMRs approximately 3.4 times those of states in the lower 10th percentile (Georgia, Louisiana, Massachusetts, Nevada, Connecticut, and New York) (Figure 4, Supporting Information S1: Table 8). By region, the West (31.24) and Midwest (30.57) had the highest pooled AAMRs, followed by the South (24.03) and Northeast (18.75), and the West had the steepest long‐term increase (AAPC 4.00%) (Supporting Information S1: Figure 3, Tables 3, 7). Rates were consistently higher in non‐metropolitan than in metropolitan areas (1999–2020 AAMR, 30.89 vs. 22.59). From 1999 to 2005, AAMRs rose significantly in both non‐metropolitan and metropolitan areas (APCs 3.71% and 3.83%, respectively). Thereafter, non‐metropolitan AAMRs declined significantly until 2015 (APC −1.73%), whereas metropolitan AAMRs remained stable until 2014 (APC −1.09%). Subsequently, AAMRs rose sharply in both non‐metropolitan and metropolitan areas until 2020 (APCs 9.31% and 7.38%, respectively) (Figure 5, Supporting Information S1: Tables 3, 6).

**Figure 4:**
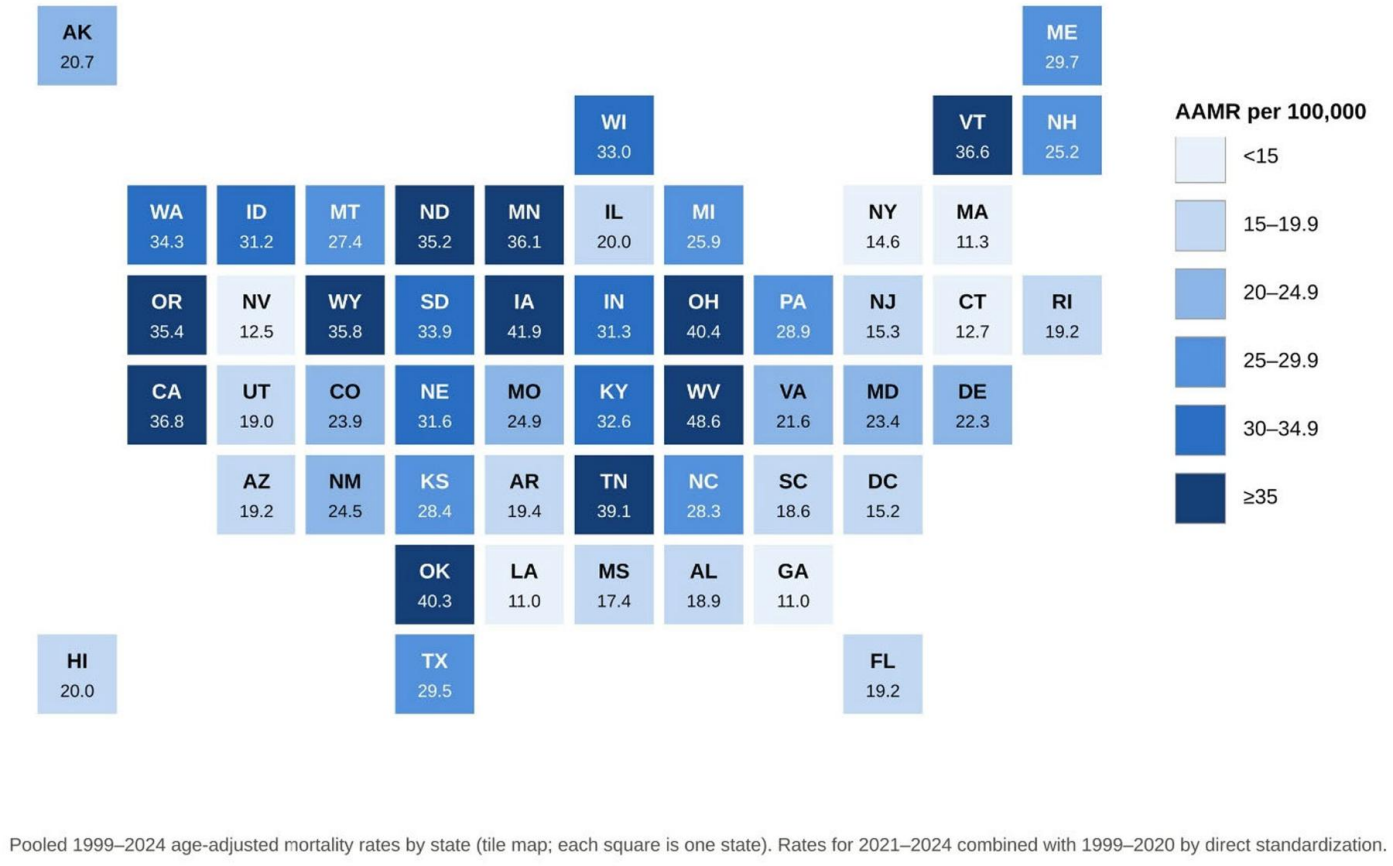
Pooled T2DM and IHD‐related age‐adjusted mortality rates per 100 000 by state among adults aged 45 and above in the United States, 1999 to 2024 (tile map; each square represents one state).

**Figure 5:**
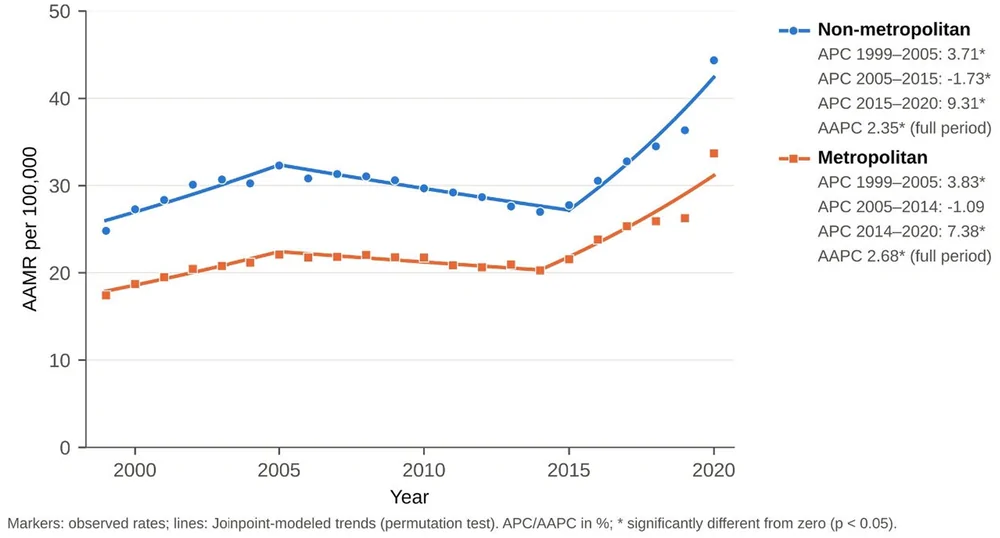
Trends in T2DM and IHD‐related age‐adjusted mortality rates per 100 000, stratified by urbanization among adults aged 45 and above in the United States, 1999 to 2020. Markers show observed rates; lines show Joinpoint‐modeled trends (permutation test). *Indicates that the APC or AAPC is significantly different from zero at *α* = 0.05. AAPC, average annual percent change; APC, annual percent change.

## 4 Discussion

Our examination of 26 years of mortality data from the CDC WONDER database highlights crucial insights. From 1999 to 2024, T2DM and IHD were co‐listed on 828 250 death certificates, and the AAMR increased overall (AAPC 2.06%), with a decline between 2005 and 2015, a steep rise until the 2021 peak, and a significant decline thereafter. Males represented 58.5% of deaths and had nearly double the AAMR of females. NH White individuals accounted for most deaths (76.9%), reflecting their population size, whereas NH American Indian or Alaska Native adults had the highest AAMR. Adults aged 75–84 years accounted for the largest share of deaths (33.5%), but age‐specific mortality rates were highest among those aged ≥ 85 years. Although metropolitan areas accounted for most deaths (77.6%), reflecting their larger population, AAMRs were consistently higher in non‐metropolitan areas. Medical facilities were the most common place of death overall (38.0%), whereas the decedent's home was the most common single location (34.8%). Lastly, West Virginia exhibited the highest AAMRs among the states.

Our analysis indicates that overall mortality from T2DM and IHD has risen despite a noticeable improvement during the intervening period. This finding contrasts with the research by Schmitt et al., which revealed a decrease in mortality rates among patients with diabetes and MI. Such improvements have primarily been attributed to advancements in medical management, the use of cardioprotective treatments like statins and antihypertensives, enhanced rates of percutaneous coronary intervention (PCI), better early detection and treatment of DM, as well as stronger public health policies and patient education [17]. Similarly, Bashar et al. reported a 29.2% fall in the diabetes‐related cardiovascular AAMR between 1999 and 2019, largely driven by a 41% decrease in IHD deaths [10]. However, a national analysis of diabetes‐ and cardiovascular disease‐related mortality found that this decline reversed after 2014 [18], consistent with the rise we observed from 2015 onward.

In our study, we observed a peak rise in AAMRs through 2021 when the COVID‐19 pandemic occurred. These results are consistent with several large studies and meta‐analyses, which demonstrate that both T2DM and IHD independently increase the risk of COVID‐19 mortality, which is further increased by their co‐existence [19, 20, 21]. Bashar et al. estimated 16 009 excess diabetes‐related cardiovascular deaths in the pandemic's first year, of which 8504 were due to IHD, and reported a 14.1% rise in diabetes‐related IHD mortality in the United States during 2020 compared with the previous year [10]. This systemic interaction between DM and COVID‐19 can possibly increase IHD mortality, as hyperglycemia and insulin resistance can aggravate virus‐induced endothelial dysfunction and inflammation, leading to plaque rupture and thrombosis [19]. Further, the use of steroid therapy was common in severe COVID‐19, which might exacerbate the inflammatory response posed by the virus and worsen glycemic control and ischemic outcomes [22, 23, 24]. Together, these factors may explain the sharp increase in IHD‐related mortality among diabetic patients observed during the pandemic period. The significant decline after 2021 across most subgroups likely reflects waning pandemic mortality, although the AAMR in 2024 remained well above its pre‐pandemic level (32.97 vs. 27.93 in 2019).

The rise in AAMRs from 2015 to 2019, before the pandemic, deserves attention because it coincided with the introduction of SGLT2 inhibitors and GLP‐1RAs, which reduced cardiovascular events and mortality in outcome trials. Several factors may explain why these benefits were not yet evident at the population level. First, uptake was slow: in 2018, fewer than 10% of insured US adults with T2DM and atherosclerotic cardiovascular disease used either drug class, and fewer than 5% received all recommended cardioprotective therapies [25, 26]. Even in cardiology clinics enrolled in a trial between 2019 and 2022, baseline use was only 12%–15% [27]. Second, access was unequal: use was lower among Medicare beneficiaries than among commercially insured patients [28], and lower among Black patients and women [29], groups that carry a substantial share of the burden in our data, pointing to cost, insurance coverage, and prescribing inertia. Third, these agents were endorsed for cardiovascular risk reduction in major guidelines only around 2018–2019 [30], so any population‐level mortality effect would be expected to lag. Finally, the rising prevalence of diabetes and obesity and longer survival with established IHD have likely enlarged the at‐risk population [1]. Because death certificates cannot be linked to medication use, these explanations remain hypotheses, and whether the decline after 2021 partly reflects broader uptake of these agents warrants study with linked clinical data.

Sex disparities continue to be a significant factor in IHD mortality associated with DM. Women with T2DM have a 40%–50% higher relative risk of cardiovascular mortality than men with T2DM, as DM seems to negate the typical cardiovascular protection advantage seen in females [31, 32]. This increased vulnerability can be attributed to the reduction of estrogen's protective effects on blood vessels, unfavorable lipid and inflammatory profiles, and more pronounced endothelial dysfunction under hyperglycemic conditions [33, 34]. Furthermore, diagnostic and treatment biases, including uncommon symptom manifestation, late diagnosis, and the underutilization of cardioprotective treatments, all worsen the outcomes in women [31]. However, we observed higher absolute mortality in men in our study, which is consistent with cardiovascular mortality trends reported across global, national, and regional literature, where age‐adjusted mortality in men exceeded that in women by 80% [35]. This is likely because IHD has a higher initial prevalence in men, who have a greater accumulation of risk factors such as smoking and hypertension [36]. Consequently, despite the increased relative risk of women after developing DM, men continue to contribute more to the total mortality burden, and therefore, it is necessary to come up with sex‐specific prevention and management strategies.

From racial and ethnic groups, NH White individuals formed the largest number of total deaths, reflecting their population size and older age structure. However, after adjusting these mortalities for age, the burden shifted to minority groups, particularly NH American Indian or Alaska Native adults in our study, highlighting a disproportionate risk considering the population sizes of subgroups [37]. Hispanic adults had the second‐highest AAMR and the steepest rise from 2017 to 2020 (APC 15.61%). Such discrepancies might occur due to factors including socioeconomic disadvantage, lack of healthcare access, increased prevalence of comorbid conditions, and structural inequities in prevention and treatment, including lower access to newer cardioprotective therapies [29], suggesting the creation of specific interventions and resources based on both absolute and AAMR [38, 39].

Although adults aged 75–84 years accounted for the largest share of deaths, age‐specific mortality rates increased steeply with age and were highest among those aged ≥ 85 years. Notably, adults aged 45–54 years had the steepest increase from 2015 to 2021 (APC 11.87%) and the highest AAPC (4.06%). Quinones et al. similarly found a disproportionate IHD mortality burden associated with diabetes in younger age groups, especially those less than 50 years of age, and particularly in women. This increased risk could be attributed to higher case fatality rates for DM in younger age groups and the fact that older persons are more likely to die from other causes before IHD. Further, younger adults generally have a low risk for developing IHD; this risk significantly increases with DM. Notably, women under 60 years of age who suffer from DM were found to have similar IHD risks as men, which is uncommon, as premenopausal females have a lower risk of IHD when considering the broader population [40].

Research shows that rural regions have a higher death rate from IHD, especially MI. This is likely related to a lack of cardiologists and emergency services, limited resources, and trained staff in these regions compared to metropolitan areas. Other factors that contribute to this include lower levels of physical activity, higher obesity, and tobacco use in rural areas compared to urban areas [41, 42]. Furthermore, peak mortality rates were observed in the West and Midwest regions. The analysis by Jain et al. also showed higher mortality associated with DM and cardiovascular disease in the Midwest and Western regions. These variations can be attributed to higher prevalence of chronic disease, economic hardships, limited healthcare services, and limited access to preventive and specialist care. Furthermore, state‐level differences in population demographics, disparities in inherent risk of diseases, and different metabolic, behavioral, and clinical risk profiles of the population may also explain these mortality variations. However, increased healthcare funding and the expansion of state‐based Medicaid could be essential to manage this mortality burden [18]. At the state level, West Virginia, Iowa, Ohio, Oklahoma, and Tennessee had the highest AAMRs. The very low rates observed in some states, such as Georgia and Louisiana, may partly reflect differences in death certificate completion and coding practices, including the use of unspecified diabetes codes, rather than true differences in mortality alone [43].

Lastly, medical facilities were the most common place of death overall, although the decedent's home was the most common single location (34.8%), which may reflect sudden cardiac deaths and an increasing preference for home‐based end‐of‐life care. In‐hospital mortality is associated with patient severity, age, sex, comorbidities, hospital ownership, size, location, teaching status, financial resources, the presence of coronary care units, and board certification of attending physicians [44].

### 4.1 Study Limitations

This study has notable limitations, primarily due to its retrospective design. The dependency on death certificates from the CDC WONDER database introduces concerns over potential misdiagnoses, leading to misclassification bias. Diabetes is under‐reported on death certificates [43], and deaths coded as unspecified diabetes (E14) were not counted, so the true burden is likely underestimated; state differences in coding practices may also contribute to the geographic variation observed. These data do not clarify whether individuals died due to DM and IHD or had these conditions at the time of death. The change from bridged‐race to single‐race categories in 2021 may affect the comparability of race‐specific estimates, and the COVID‐19 pandemic may have altered cause‐of‐death reporting in 2020–2022. Urbanization‐specific rates were available only through 2020. The final Joinpoint segment (2021–2024) is short and should be interpreted with caution, although the permutation test and WBIC selected identical models for 17 of 19 series. Furthermore, the absence of laboratory data, medication histories, disease severity, and clinical insights on overall health, comorbidities, and treatments hinders a thorough analysis of mortality trends. Nevertheless, by focusing specifically on IHD and including adults aged 45 years and older, this study complements recent analyses restricted to older adults or to all cardiovascular causes [7], and provides a 26‐year view of IHD mortality in T2DM that spans the period before the introduction of SGLT2 inhibitors and GLP‐1RAs, the COVID‐19 pandemic, and its aftermath. Future studies utilizing clinical registries linked to treatment data might provide more nuanced insights.

## 5 Conclusions

The analysis of 26 years of mortality data shows that mortality rates associated with T2DM and IHD increased from 1999 to 2024, rising steeply from 2015, peaking in 2021 during the COVID‐19 pandemic, and declining thereafter while remaining above pre‐pandemic levels. The recorded rates were highest among males, NH American Indian or Alaska Native adults, older adults, non‐metropolitan regions, and the West and Midwest regions. Our findings emphasize that more effective and equitable resource allocation, including equitable access to cardioprotective therapies, and early and specified intervention approaches for high‐risk populations are needed, together with the establishment of real‐time national surveillance systems with improvements in chronic disease registries.

## Author Contributions

Muhammad Shaheer Bin Faheem and Syed Tawassul Hassan contributed to conceptualization, writing the original draft, writing, review and editing, data curation, visualization, software, formal analysis, and project administration. Syed Atta Ur Rafe contributed to methodology, writing the original draft, writing, review and editing, investigation, visualization, and project administration. Sivaram Neppala contributed to validation, writing the original draft, supervision, and resources. Nafila Zeeshan contributed to methodology and resources. Akshay Kumar and Nimra Shafi contributed to writing, review and editing, and validation. Sumaya Samadi contributed to methodology and resources. Yasar Sattar contributed to supervision and project administration.

## Funding

The authors have nothing to report.

## Ethics Statement

This study used publicly available, de‐identified data from the Centers for Disease Control and Prevention Wide‐ranging Online Data for Epidemiologic Research (CDC WONDER) database and did not involve human participants or identifiable private information. As such, institutional ethics approval and informed consent were not required, in accordance with national regulations and institutional policies. The study complies with the ethical standards of the Declaration of Helsinki.

## Consent

The authors have nothing to report.

## Conflicts of Interest

The authors declare no conflicts of interest.

## Supporting information


Supporting File

## Data Availability

The data sets generated during and/or analyzed during the current study are publicly available from CDC WONDER; all query parameters and saved query URLs are provided in Supporting Information S1: Table 9.
