# Supplementary material for: Temporal Trends and Disparities in Mortality Associated With Type 2 Diabetes and Ischemic Heart Disease in the United States (1999−2024)
Source: Clin Cardiol. 2026 Nov 5;49(11):e70513. doi: 10.1002/clc.70513 (PMC13739127; doi:10.1002/clc.70513)
Supplement: Supplementary file 1 [file CLC-49-e70513-s001.docx]

**Supplementary Material**

**Temporal Trends and Disparities in Mortality Associated with Type 2 Diabetes and Ischemic Heart Disease in the United States (1999-2024)**

Supplemental Table 1. Deaths by demographic and geographic characteristics, 1999–2024

Supplementary Figure 1. Place of death, 1999–2024

Supplemental Table 2. Overall and sex-stratified AAMRs, 1999–2024

Supplementary Figure 2A/2B. Age-specific mortality rate trends (45–64 and ≥65 years)

Supplemental Table 3. Annual percent change (APC) and average annual percent change (AAPC)

Supplemental Table 4. Race- and ethnicity-stratified AAMRs, 1999–2024

Supplemental Table 5. Age-specific mortality rates, 1999–2024

Supplemental Table 6. Urbanization-stratified AAMRs, 1999–2020

Supplemental Table 7. Census region-stratified AAMRs, 1999–2024

Supplementary Figure 3. Census region trends, 1999–2024

Supplemental Table 8. State-level pooled AAMRs, 1999–2024

Supplemental Table 9. CDC WONDER query parameters and saved query URLs

Supplemental Table 10. Joinpoint settings and permutation test results for model selection

Supplemental Table 11. Sensitivity analysis: permutation test vs weighted BIC model selection

*Abbreviations: AAMR, age-adjusted mortality rate per 100,000; CI, confidence interval; NH, non-Hispanic; APC, annual percent change; AAPC, average annual percent change; WBIC, weighted Bayesian information criterion.*

**Supplemental Table 1.** Absolute number of T2DM and IHD-related deaths and percentage of total deaths among adults aged 45 and above, stratified by sex, race and ethnicity, age group, place of death, urbanization, and census region in the United States, 1999–2024.

| **Characteristic** | **Deaths** | **% of total deaths** |
| --- | --- | --- |
| **Overall** | **828,250** | **100.0** |
| **Sex** |  |  |
| Female | 343,392 | 41.5 |
| Male | 484,858 | 58.5 |
| **Race and ethnicity** |  |  |
| NH American Indian or Alaska Native | 7,650 | 0.9 |
| NH Asian or Pacific Islander | 27,289 | 3.3 |
| NH Black or African American | 78,493 | 9.5 |
| NH White | 636,930 | 76.9 |
| Hispanic or Latino | 75,066 | 9.1 |
| Other or unknown race/ethnicity* | 2,822 | 0.3 |
| **Age group** |  |  |
| 45-54 years | 29,859 | 3.6 |
| 55-64 years | 100,988 | 12.2 |
| 65-74 years | 203,229 | 24.5 |
| 75-84 years | 277,862 | 33.5 |
| 85+ years | 216,312 | 26.1 |
| **Place of death** |  |  |
| Medical facility – inpatient | 218,149 | 26.3 |
| Medical facility – outpatient or ER | 89,883 | 10.9 |
| Medical facility – dead on arrival | 5,976 | 0.7 |
| Medical facility – status unknown | 345 | 0.0 |
| Decedent’s home | 288,310 | 34.8 |
| Hospice facility | 21,979 | 2.7 |
| Nursing home/long-term care | 171,414 | 20.7 |
| Other | 31,084 | 3.8 |
| Place of death unknown | 1,110 | 0.1 |
| **Urbanization** |  |  |
| Metropolitan | 642,644 | 77.6 |
| Non-metropolitan | 185,606 | 22.4 |
| **Census region** |  |  |
| Northeast | 116,871 | 14.1 |
| Midwest | 217,287 | 26.2 |
| South | 280,178 | 33.8 |
| West | 213,914 | 25.8 |

NH: non-Hispanic. *Decedents with Hispanic origin not stated, or (2021–2024) more than one race or race not available. Urbanization counts combine 1999–2020 (bridged-race file) and 2021–2024 (single-race file) death counts; urbanization-specific rates are available only for 1999–2020. Percentages may not sum to 100 because of rounding.

**Supplementary Figure 1.** Percentage of total T2DM and IHD-related deaths by place of death among adults aged 45 and above in the United States, 1999 to 2024.

**Supplemental Table 2.** Overall and sex-stratified T2DM and IHD-related age-adjusted mortality rates per 100,000 (95% CI) among adults aged 45 and above in the United States, 1999 to 2024.

| **Year** | **Overall** | **Male** | **Female** |
| --- | --- | --- | --- |
| 1999 | 18.87 (18.59–19.15) | 24.16 (23.65–24.66) | 15.30 (14.98–15.62) |
| 2000 | 20.40 (20.11–20.68) | 25.80 (25.28–26.32) | 16.70 (16.37–17.04) |
| 2001 | 21.15 (20.87–21.44) | 26.94 (26.42–27.47) | 17.26 (16.93–17.60) |
| 2002 | 22.27 (21.98–22.56) | 28.90 (28.36–29.44) | 17.87 (17.53–18.21) |
| 2003 | 22.62 (22.32–22.91) | 29.80 (29.26–30.34) | 17.73 (17.39–18.06) |
| 2004 | 22.90 (22.60–23.19) | 30.02 (29.48–30.56) | 17.94 (17.60–18.27) |
| 2005 | 23.94 (23.65–24.24) | 31.47 (30.93–32.02) | 18.76 (18.42–19.10) |
| 2006 | 23.46 (23.17–23.75) | 31.06 (30.53–31.59) | 18.02 (17.69–18.35) |
| 2007 | 23.53 (23.24–23.82) | 31.01 (30.48–31.53) | 18.17 (17.84–18.50) |
| 2008 | 23.68 (23.39–23.96) | 31.55 (31.02–32.07) | 18.06 (17.74–18.39) |
| 2009 | 23.37 (23.09–23.65) | 31.58 (31.06–32.10) | 17.45 (17.13–17.77) |
| 2010 | 23.17 (22.89–23.45) | 31.21 (30.70–31.72) | 17.31 (16.99–17.63) |
| 2011 | 22.33 (22.06–22.61) | 30.41 (29.92–30.91) | 16.45 (16.14–16.76) |
| 2012 | 22.07 (21.81–22.34) | 30.59 (30.10–31.08) | 15.86 (15.56–16.15) |
| 2013 | 22.12 (21.86–22.38) | 30.53 (30.05–31.01) | 15.91 (15.61–16.20) |
| 2014 | 21.46 (21.20–21.71) | 30.11 (29.64–30.58) | 14.96 (14.67–15.24) |
| 2015 | 22.62 (22.36–22.88) | 31.62 (31.14–32.09) | 15.88 (15.59–16.17) |
| 2016 | 24.96 (24.69–25.23) | 35.07 (34.58–35.57) | 17.28 (16.99–17.58) |
| 2017 | 26.59 (26.32–26.87) | 37.48 (36.98–37.98) | 18.33 (18.03–18.64) |
| 2018 | 27.37 (27.09–27.64) | 39.13 (38.62–39.63) | 18.36 (18.06–18.66) |
| 2019 | 27.93 (27.65–28.20) | 39.87 (39.36–40.37) | 18.78 (18.48–19.09) |
| 2020 | 35.45 (35.14–35.75) | 50.82 (50.26–51.39) | 23.65 (23.32–23.99) |
| 2021 | 37.65 (37.33–37.97) | 54.14 (53.56–54.74) | 24.85 (24.50–25.20) |
| 2022 | 35.21 (34.91–35.51) | 51.14 (50.58–51.71) | 23.15 (22.82–23.48) |
| 2023 | 33.24 (32.95–33.54) | 48.23 (47.70–48.78) | 21.65 (21.33–21.97) |
| 2024 | 32.97 (32.68–33.26) | 47.65 (47.12–48.18) | 21.52 (21.21–21.83) |
| **Pooled 1999–2024** | **26.03 (25.98–26.09)** | **36.33 (36.23–36.44)** | **18.53 (18.47–18.59)** |

Pooled rates were calculated by direct standardization to the 2000 U.S. standard population from ten-year age-specific deaths and populations summed across the 1999–2020 and 2018–2024 CDC WONDER files.

**Supplementary Figure 2A.** Trends in T2DM and IHD-related age-specific mortality rates among adults aged 45–64 years in the United States, 1999 to 2024.

Markers show observed rates; lines show Joinpoint-modeled trends (permutation test). APC = Annual Percent Change; AAPC = Average Annual Percent Change. *Significantly different from zero at α = 0.05.

**Supplementary Figure 2B.** Trends in T2DM and IHD-related age-specific mortality rates among adults aged 65 years and above in the United States, 1999 to 2024.

Markers show observed rates; lines show Joinpoint-modeled trends (permutation test). APC = Annual Percent Change; AAPC = Average Annual Percent Change. *Significantly different from zero at α = 0.05.

**Supplemental Table 3.** Annual percent change (APC) and average annual percent change (AAPC) of T2DM and IHD-related age-adjusted mortality rates (age-specific rates for age groups) among adults aged 45 and above in the United States, 1999 to 2024.

| **Group** | **Period** | **Measure** | **Estimate, % (95% CI)** | **P value** |
| --- | --- | --- | --- | --- |
| **Overall** |  |  |  |  |
| Overall | 1999–2005 | APC | 3.51* (1.52 to 8.78) | <0.001 |
|  | 2005–2015 | APC | -0.93* (-4.39 to -0.05) | 0.041 |
|  | 2015–2021 | APC | 9.06* (7.35 to 12.89) | <0.001 |
|  | 2021–2024 | APC | -4.04* (-7.57 to -0.46) | 0.025 |
|  | 1999–2024 | AAPC | 2.06* (1.68 to 2.61) | <0.001 |
| **Sex** |  |  |  |  |
| Female | 1999–2005 | APC | 2.76* (0.91 to 6.57) | 0.001 |
|  | 2005–2015 | APC | -2.06* (-4.28 to -1.20) | <0.001 |
|  | 2015–2021 | APC | 7.99* (6.16 to 11.96) | <0.001 |
|  | 2021–2024 | APC | -4.38* (-8.62 to -0.72) | 0.017 |
|  | 1999–2024 | AAPC | 1.13* (0.76 to 1.59) | <0.001 |
| Male | 1999–2004 | APC | 4.92* (2.06 to 14.37) | <0.001 |
|  | 2004–2015 | APC | -0.11 (-3.92 to 0.68) | 0.722 |
|  | 2015–2021 | APC | 9.51* (7.80 to 13.63) | <0.001 |
|  | 2021–2024 | APC | -3.84* (-7.46 to -0.21) | 0.037 |
|  | 1999–2024 | AAPC | 2.66* (2.25 to 3.32) | <0.001 |
| **Race and ethnicity** |  |  |  |  |
| NH American Indian or Alaska Native | 1999–2003 | APC | 10.31* (3.12 to 30.06) | <0.001 |
|  | 2003–2014 | APC | -0.95 (-8.18 to 0.61) | 0.208 |
|  | 2014–2021 | APC | 7.12* (4.81 to 14.33) | <0.001 |
|  | 2021–2024 | APC | -6.78* (-15.11 to -1.59) | 0.012 |
|  | 1999–2024 | AAPC | 2.26* (1.49 to 3.47) | <0.001 |
| NH Asian or Pacific Islander | 1999–2014 | APC | 2.79* (1.30 to 4.00) | 0.006 |
|  | 2014–2021 | APC | 9.77* (8.02 to 14.20) | <0.001 |
|  | 2021–2024 | APC | -7.37* (-11.48 to -2.99) | 0.001 |
|  | 1999–2024 | AAPC | 3.40* (2.94 to 3.99) | <0.001 |
| NH Black or African American | 1999–2006 | APC | 3.76* (1.78 to 9.18) | <0.001 |
|  | 2006–2014 | APC | -2.21* (-7.84 to -0.53) | 0.010 |
|  | 2014–2021 | APC | 7.56* (5.84 to 12.30) | <0.001 |
|  | 2021–2024 | APC | -4.26* (-10.06 to -0.30) | 0.037 |
|  | 1999–2024 | AAPC | 1.85* (1.41 to 2.46) | <0.001 |
| NH White | 1999–2005 | APC | 3.53* (1.70 to 7.26) | <0.001 |
|  | 2005–2015 | APC | -1.19* (-3.23 to -0.34) | 0.011 |
|  | 2015–2021 | APC | 8.98* (7.31 to 12.61) | <0.001 |
|  | 2021–2024 | APC | -3.05 (-6.54 to 0.33) | 0.076 |
|  | 1999–2024 | AAPC | 2.07* (1.71 to 2.52) | <0.001 |
| Hispanic or Latino | 1999–2017 | APC | 1.60* (0.55 to 2.44) | 0.013 |
|  | 2017–2020 | APC | 15.61* (8.39 to 19.04) | <0.001 |
|  | 2020–2024 | APC | -4.76* (-8.83 to -2.31) | 0.004 |
|  | 1999–2024 | AAPC | 2.13* (1.61 to 2.64) | <0.001 |
| **Age group (age-specific rates)** |  |  |  |  |
| 45–54 years | 1999–2003 | APC | 7.20* (3.16 to 19.20) | <0.001 |
|  | 2003–2015 | APC | 1.36 (-2.03 to 2.05) | 0.097 |
|  | 2015–2021 | APC | 11.87* (10.13 to 15.73) | <0.001 |
|  | 2021–2024 | APC | -3.86 (-7.49 to 0.25) | 0.066 |
|  | 1999–2024 | AAPC | 4.06* (3.59 to 4.80) | <0.001 |
| 55–64 years | 1999–2006 | APC | 2.83* (1.07 to 9.24) | 0.002 |
|  | 2006–2014 | APC | -1.55* (-7.03 to -0.08) | 0.044 |
|  | 2014–2021 | APC | 9.58* (7.89 to 12.84) | <0.001 |
|  | 2021–2024 | APC | -4.07* (-8.42 to -0.22) | 0.034 |
|  | 1999–2024 | AAPC | 2.37* (1.94 to 3.01) | <0.001 |
| 65–74 years | 1999–2005 | APC | 3.42* (1.52 to 7.86) | <0.001 |
|  | 2005–2014 | APC | -1.76* (-4.56 to -0.73) | 0.003 |
|  | 2014–2021 | APC | 7.92* (6.67 to 10.09) | <0.001 |
|  | 2021–2024 | APC | -5.55* (-8.61 to -2.09) | 0.001 |
|  | 1999–2024 | AAPC | 1.63* (1.29 to 2.12) | <0.001 |
| 75–84 years | 1999–2005 | APC | 3.64* (1.69 to 8.28) | <0.001 |
|  | 2005–2015 | APC | -1.08* (-4.03 to -0.13) | 0.026 |
|  | 2015–2021 | APC | 8.38* (6.57 to 12.68) | <0.001 |
|  | 2021–2024 | APC | -4.50* (-8.24 to -0.92) | 0.010 |
|  | 1999–2024 | AAPC | 1.82* (1.44 to 2.32) | <0.001 |
| 85+ years | 1999–2005 | APC | 3.75* (1.49 to 9.33) | <0.001 |
|  | 2005–2015 | APC | -1.00* (-4.53 to -0.05) | 0.044 |
|  | 2015–2021 | APC | 8.99* (7.25 to 13.88) | <0.001 |
|  | 2021–2024 | APC | -1.36 (-6.74 to 2.10) | 0.395 |
|  | 1999–2024 | AAPC | 2.41* (1.98 to 3.00) | <0.001 |
| **Urbanization (1999–2020)** |  |  |  |  |
| Metropolitan | 1999–2005 | APC | 3.83* (1.62 to 10.97) | <0.001 |
|  | 2005–2014 | APC | -1.09 (-5.60 to 0.13) | 0.068 |
|  | 2014–2020 | APC | 7.38* (5.42 to 10.85) | 0.001 |
|  | 1999–2020 | AAPC | 2.68* (2.22 to 3.37) | <0.001 |
| Non-metropolitan | 1999–2005 | APC | 3.71* (2.23 to 6.14) | <0.001 |
|  | 2005–2015 | APC | -1.73* (-2.84 to -1.04) | <0.001 |
|  | 2015–2020 | APC | 9.31* (7.54 to 12.12) | <0.001 |
|  | 1999–2020 | AAPC | 2.35* (2.04 to 2.74) | <0.001 |
| **Census region** |  |  |  |  |
| Northeast | 1999–2003 | APC | 3.36 (-0.15 to 10.98) | 0.065 |
|  | 2003–2015 | APC | -2.43* (-4.67 to -1.71) | 0.005 |
|  | 2015–2021 | APC | 8.49* (6.56 to 12.96) | <0.001 |
|  | 2021–2024 | APC | -5.03* (-11.01 to -0.85) | 0.017 |
|  | 1999–2024 | AAPC | 0.69* (0.25 to 1.18) | 0.006 |
| Midwest | 1999–2005 | APC | 3.63* (1.53 to 7.88) | <0.001 |
|  | 2005–2014 | APC | -2.50* (-5.67 to -1.34) | <0.001 |
|  | 2014–2021 | APC | 7.76* (6.18 to 11.75) | <0.001 |
|  | 2021–2024 | APC | -3.59 (-8.91 to 0.09) | 0.055 |
|  | 1999–2024 | AAPC | 1.61* (1.17 to 2.14) | <0.001 |
| South | 1999–2005 | APC | 3.75* (1.83 to 7.31) | <0.001 |
|  | 2005–2015 | APC | -2.02* (-3.58 to -1.16) | <0.001 |
|  | 2015–2021 | APC | 9.72* (8.15 to 12.54) | <0.001 |
|  | 2021–2024 | APC | -3.51* (-6.72 to -0.08) | 0.044 |
|  | 1999–2024 | AAPC | 1.88* (1.55 to 2.32) | <0.001 |
| West | 1999–2008 | APC | 5.44* (4.33 to 12.14) | <0.001 |
|  | 2008–2014 | APC | 1.22 (-4.20 to 3.32) | 0.492 |
|  | 2014–2021 | APC | 8.05* (6.62 to 12.69) | <0.001 |
|  | 2021–2024 | APC | -3.64* (-7.74 to -0.20) | 0.040 |
|  | 1999–2024 | AAPC | 4.00* (3.61 to 4.73) | <0.001 |

Joinpoint Regression Program version 5.2.0; log-linear model; heteroscedastic errors (CDC WONDER standard errors); maximum of four joinpoints; number of joinpoints selected by permutation test (4,499 permutations, overall α = 0.05); 95% CIs by the empirical quantile method. *Significantly different from zero (P < 0.05). NH: non-Hispanic.

**Supplemental Table 4.** Race- and ethnicity-stratified T2DM and IHD-related age-adjusted mortality rates per 100,000 (95% CI) among adults aged 45 and above in the United States, 1999 to 2024.

| **Year** | **NH American Indian or Alaska Native** | **NH Asian or Pacific Islander** | **NH Black or African American** | **NH White** | **Hispanic or Latino** |
| --- | --- | --- | --- | --- | --- |
| 1999 | 32.06 (26.06–38.06) | 11.48 (9.95–13.01) | 19.91 (18.91–20.90) | 18.75 (18.45–19.05) | 20.56 (19.17–21.95) |
| 2000 | 28.10 (22.80–33.41) | 11.23 (9.78–12.67) | 21.24 (20.22–22.27) | 20.35 (20.04–20.66) | 20.72 (19.36–22.08) |
| 2001 | 32.40 (26.66–38.14) | 12.52 (11.03–14.00) | 22.19 (21.15–23.22) | 21.11 (20.80–21.43) | 22.07 (20.71–23.43) |
| 2002 | 35.10 (29.31–40.89) | 13.87 (12.35–15.39) | 24.29 (23.21–25.37) | 22.17 (21.85–22.50) | 22.55 (21.21–23.88) |
| 2003 | 46.38 (39.65–53.11) | 12.43 (11.06–13.81) | 23.87 (22.81–24.93) | 22.64 (22.32–22.96) | 22.72 (21.40–24.03) |
| 2004 | 36.64 (30.81–42.47) | 13.12 (11.76–14.49) | 23.60 (22.55–24.65) | 22.97 (22.65–23.29) | 22.42 (21.16–23.69) |
| 2005 | 43.55 (37.27–49.82) | 13.04 (11.73–14.36) | 25.81 (24.73–26.89) | 23.82 (23.49–24.14) | 25.71 (24.40–27.02) |
| 2006 | 41.92 (35.97–47.87) | 13.66 (12.35–14.97) | 26.74 (25.65–27.83) | 23.08 (22.76–23.40) | 26.24 (24.93–27.54) |
| 2007 | 41.45 (35.56–47.34) | 14.90 (13.57–16.23) | 25.61 (24.55–26.67) | 23.35 (23.03–23.67) | 25.68 (24.42–26.93) |
| 2008 | 36.91 (31.50–42.33) | 15.86 (14.52–17.20) | 24.76 (23.74–25.78) | 23.59 (23.27–23.90) | 25.35 (24.14–26.56) |
| 2009 | 40.92 (35.21–46.62) | 15.84 (14.54–17.13) | 25.38 (24.36–26.40) | 22.98 (22.66–23.29) | 26.90 (25.69–28.11) |
| 2010 | 37.25 (31.91–42.59) | 16.05 (14.77–17.32) | 24.67 (23.67–25.67) | 22.78 (22.47–23.09) | 27.21 (26.02–28.41) |
| 2011 | 37.09 (32.08–42.11) | 15.46 (14.25–16.67) | 23.54 (22.58–24.50) | 22.12 (21.82–22.42) | 25.43 (24.31–26.54) |
| 2012 | 40.20 (34.98–45.43) | 15.36 (14.20–16.51) | 23.26 (22.32–24.19) | 21.79 (21.49–22.08) | 25.07 (23.99–26.15) |
| 2013 | 39.29 (34.27–44.32) | 17.92 (16.71–19.13) | 23.72 (22.80–24.65) | 21.54 (21.24–21.83) | 27.00 (25.92–28.09) |
| 2014 | 37.31 (32.57–42.04) | 16.50 (15.38–17.62) | 22.31 (21.43–23.19) | 20.90 (20.61–21.19) | 26.13 (25.10–27.17) |
| 2015 | 41.61 (36.72–46.50) | 20.29 (19.09–21.49) | 23.62 (22.72–24.51) | 21.92 (21.63–22.21) | 27.32 (26.30–28.34) |
| 2016 | 43.42 (38.54–48.31) | 21.31 (20.12–22.50) | 27.06 (26.12–28.00) | 24.21 (23.91–24.51) | 29.22 (28.18–30.25) |
| 2017 | 48.02 (43.04–53.00) | 23.95 (22.73–25.17) | 27.07 (26.15–27.99) | 25.80 (25.49–26.11) | 32.05 (30.99–33.10) |
| 2018 | 44.20 (39.63–48.77) | 25.56 (24.33–26.80) | 28.05 (27.13–28.97) | 26.54 (26.23–26.85) | 32.04 (31.01–33.06) |
| 2019 | 46.98 (42.35–51.61) | 24.88 (23.70–26.07) | 28.31 (27.40–29.22) | 27.20 (26.88–27.51) | 33.23 (32.20–34.25) |
| 2020 | 62.62 (57.39–67.86) | 33.10 (31.77–34.43) | 37.40 (36.37–38.44) | 33.70 (33.35–34.05) | 46.39 (45.21–47.57) |
| 2021 | 62.33 (57.05–68.01) | 33.54 (32.19–34.92) | 37.93 (36.87–39.01) | 36.74 (36.38–37.12) | 45.29 (44.13–46.46) |
| 2022 | 54.49 (49.69–59.65) | 30.81 (29.58–32.09) | 35.02 (34.03–36.04) | 34.79 (34.44–35.14) | 40.52 (39.45–41.60) |
| 2023 | 53.48 (48.81–58.52) | 28.91 (27.74–30.12) | 33.68 (32.71–34.66) | 32.71 (32.37–33.05) | 38.02 (37.01–39.06) |
| 2024 | 49.46 (45.12–54.15) | 26.97 (25.88–28.10) | 32.76 (31.84–33.71) | 32.85 (32.51–33.18) | 36.62 (35.66–37.60) |
| **Pooled 1999–2024** | **44.27 (43.23–45.30)** | **21.83 (21.57–22.10)** | **27.34 (27.14–27.53)** | **25.37 (25.31–25.44)** | **31.26 (31.03–31.49)** |

Bridged-race categories for 1999–2020; single-race categories for 2021–2024, with NH Asian and NH Native Hawaiian or Other Pacific Islander combined. NH: non-Hispanic.

**Supplemental Table 5.** Age-specific T2DM and IHD-related mortality rates per 100,000 (95% CI) among adults aged 45 and above in the United States, 1999 to 2024.

| **Year** | **45–54 years** | **55–64 years** | **65–74 years** | **75–84 years** | **85+ years** |
| --- | --- | --- | --- | --- | --- |
| 1999 | 1.57 (1.44–1.70) | 7.82 (7.46–8.17) | 23.29 (22.59–23.98) | 56.41 (55.08–57.74) | 103.95 (100.85–107.05) |
| 2000 | 1.67 (1.54–1.80) | 8.28 (7.92–8.65) | 24.89 (24.17–25.61) | 59.93 (58.56–61.29) | 117.56 (114.29–120.82) |
| 2001 | 1.81 (1.68–1.94) | 8.13 (7.78–8.48) | 26.54 (25.79–27.28) | 62.11 (60.73–63.49) | 121.74 (118.45–125.03) |
| 2002 | 1.93 (1.79–2.06) | 8.68 (8.33–9.03) | 27.51 (26.75–28.26) | 64.87 (63.48–66.27) | 130.22 (126.83–133.60) |
| 2003 | 2.05 (1.91–2.19) | 9.03 (8.68–9.38) | 28.10 (27.34–28.87) | 66.53 (65.12–67.94) | 128.23 (124.91–131.55) |
| 2004 | 2.09 (1.96–2.23) | 8.96 (8.61–9.30) | 27.96 (27.20–28.72) | 67.46 (66.05–68.87) | 131.15 (127.82–134.48) |
| 2005 | 2.15 (2.01–2.29) | 9.60 (9.25–9.94) | 29.95 (29.17–30.73) | 70.11 (68.68–71.55) | 135.75 (132.41–139.08) |
| 2006 | 2.15 (2.01–2.29) | 9.25 (8.92–9.59) | 28.39 (27.64–29.14) | 69.91 (68.48–71.34) | 132.70 (129.46–135.93) |
| 2007 | 2.22 (2.08–2.36) | 9.21 (8.88–9.54) | 27.68 (26.95–28.42) | 69.82 (68.39–71.25) | 138.13 (134.88–141.37) |
| 2008 | 2.24 (2.10–2.38) | 9.55 (9.22–9.88) | 28.29 (27.57–29.02) | 69.71 (68.28–71.14) | 136.80 (133.62–139.98) |
| 2009 | 2.27 (2.13–2.41) | 9.30 (8.99–9.62) | 28.31 (27.60–29.03) | 69.06 (67.63–70.48) | 132.47 (129.39–135.55) |
| 2010 | 2.24 (2.10–2.37) | 9.16 (8.85–9.47) | 26.95 (26.26–27.64) | 69.27 (67.84–70.69) | 134.45 (131.39–137.52) |
| 2011 | 2.29 (2.15–2.43) | 9.03 (8.73–9.33) | 26.28 (25.61–26.95) | 67.03 (65.63–68.43) | 125.50 (122.60–128.40) |
| 2012 | 2.28 (2.14–2.42) | 8.68 (8.38–8.97) | 26.04 (25.40–26.69) | 64.07 (62.71–65.43) | 130.96 (128.04–133.88) |
| 2013 | 2.33 (2.18–2.47) | 8.77 (8.48–9.07) | 25.94 (25.31–26.57) | 65.05 (63.69–66.41) | 129.01 (126.14–131.87) |
| 2014 | 2.36 (2.21–2.50) | 8.66 (8.37–8.95) | 25.60 (24.99–26.21) | 62.68 (61.35–64.00) | 122.02 (119.26–124.78) |
| 2015 | 2.52 (2.37–2.67) | 9.24 (8.95–9.54) | 27.36 (26.74–27.97) | 65.57 (64.23–66.92) | 128.37 (125.57–131.17) |
| 2016 | 2.85 (2.69–3.01) | 10.43 (10.12–10.74) | 29.77 (29.13–30.40) | 71.81 (70.42–73.20) | 142.59 (139.66–145.52) |
| 2017 | 3.09 (2.93–3.26) | 11.13 (10.81–11.45) | 31.59 (30.95–32.23) | 77.37 (75.95–78.80) | 149.80 (146.82–152.78) |
| 2018 | 3.36 (3.18–3.54) | 11.61 (11.28–11.93) | 33.42 (32.77–34.07) | 76.92 (75.53–78.30) | 155.47 (152.45–158.49) |
| 2019 | 3.43 (3.26–3.61) | 11.95 (11.62–12.28) | 33.72 (33.08–34.36) | 78.97 (77.60–80.35) | 158.50 (155.47–161.54) |
| 2020 | 4.45 (4.24–4.65) | 15.49 (15.11–15.86) | 42.11 (41.40–42.81) | 100.81 (99.28–102.35) | 200.26 (196.86–203.66) |
| 2021 | 4.87 (4.65–5.08) | 16.70 (16.31–17.08) | 43.64 (42.94–44.35) | 105.86 (104.27–107.44) | 217.51 (213.77–221.25) |
| 2022 | 4.51 (4.30–4.72) | 15.53 (15.16–15.91) | 40.61 (39.93–41.29) | 99.23 (97.75–100.70) | 205.14 (201.65–208.62) |
| 2023 | 4.42 (4.22–4.63) | 14.42 (14.05–14.78) | 37.13 (36.49–37.78) | 91.83 (90.44–93.21) | 204.26 (200.70–207.82) |
| 2024 | 4.25 (4.05–4.45) | 14.44 (14.07–14.80) | 36.91 (36.28–37.55) | 91.52 (90.17–92.87) | 201.60 (198.13–205.07) |
| **Pooled 1999–2024** | **2.74 (2.71–2.77)** | **10.80 (10.74–10.87)** | **31.36 (31.22–31.50)** | **75.12 (74.84–75.40)** | **149.59 (148.96–150.22)** |

Age-specific (crude) mortality rates; age adjustment is not applied within age strata.

**Supplemental Table 6.** Urbanization-stratified T2DM and IHD-related age-adjusted mortality rates per 100,000 (95% CI) among adults aged 45 and above in the United States, 1999 to 2020.

| **Year** | **Metropolitan** | **Non-metropolitan** |
| --- | --- | --- |
| 1999 | 17.42 (17.13–17.72) | 24.81 (24.09–25.54) |
| 2000 | 18.75 (18.45–19.05) | 27.28 (26.52–28.04) |
| 2001 | 19.52 (19.21–19.82) | 28.34 (27.57–29.11) |
| 2002 | 20.45 (20.14–20.76) | 30.09 (29.30–30.88) |
| 2003 | 20.80 (20.49–21.11) | 30.71 (29.92–31.50) |
| 2004 | 21.18 (20.87–21.49) | 30.25 (29.47–31.03) |
| 2005 | 22.09 (21.77–22.40) | 32.31 (31.51–33.12) |
| 2006 | 21.78 (21.47–22.09) | 30.82 (30.04–31.60) |
| 2007 | 21.81 (21.50–22.12) | 31.31 (30.53–32.09) |
| 2008 | 22.06 (21.75–22.36) | 31.04 (30.27–31.81) |
| 2009 | 21.80 (21.49–22.10) | 30.61 (29.85–31.38) |
| 2010 | 21.76 (21.46–22.06) | 29.70 (28.95–30.44) |
| 2011 | 20.88 (20.59–21.17) | 29.20 (28.46–29.94) |
| 2012 | 20.65 (20.37–20.93) | 28.69 (27.97–29.41) |
| 2013 | 20.96 (20.68–21.24) | 27.61 (26.90–28.31) |
| 2014 | 20.27 (20.00–20.55) | 26.99 (26.29–27.68) |
| 2015 | 21.56 (21.28–21.84) | 27.76 (27.06–28.46) |
| 2016 | 23.81 (23.52–24.10) | 30.56 (29.83–31.29) |
| 2017 | 25.35 (25.05–25.65) | 32.78 (32.03–33.53) |
| 2018 | 25.92 (25.63–26.22) | 34.49 (33.73–35.25) |
| 2019 | 26.27 (25.98–26.56) | 36.35 (35.57–37.12) |
| 2020 | 33.71 (33.38–34.03) | 44.35 (43.50–45.20) |
| **Total 1999–2020** | **22.59 (22.52–22.65)** | **30.89 (30.73–31.05)** |

2013 NCHS Urban–Rural Classification Scheme. Population denominators for urbanization categories are not released in the single-race (2021–2024) file.

**Supplemental Table 7.** Census region-stratified T2DM and IHD-related age-adjusted mortality rates per 100,000 (95% CI) among adults aged 45 and above in the United States, 1999 to 2024.

| **Year** | **Northeast** | **Midwest** | **South** | **West** |
| --- | --- | --- | --- | --- |
| 1999 | 17.18 (16.61–17.75) | 23.90 (23.27–24.54) | 18.07 (17.61–18.52) | 15.94 (15.36–16.51) |
| 2000 | 18.60 (18.01–19.19) | 26.34 (25.67–27.00) | 19.50 (19.03–19.97) | 16.66 (16.08–17.23) |
| 2001 | 18.74 (18.15–19.34) | 26.69 (26.03–27.36) | 20.81 (20.33–21.30) | 17.76 (17.17–18.35) |
| 2002 | 19.43 (18.83–20.03) | 28.31 (27.63–28.99) | 21.65 (21.16–22.13) | 19.17 (18.56–19.78) |
| 2003 | 19.70 (19.10–20.30) | 28.86 (28.18–29.54) | 21.95 (21.46–22.44) | 19.61 (19.00–20.21) |
| 2004 | 19.66 (19.07–20.26) | 28.67 (27.99–29.35) | 21.93 (21.45–22.41) | 20.96 (20.34–21.58) |
| 2005 | 19.57 (18.98–20.16) | 30.19 (29.50–30.88) | 23.25 (22.75–23.74) | 22.45 (21.82–23.08) |
| 2006 | 18.43 (17.86–19.01) | 29.14 (28.46–29.81) | 22.73 (22.25–23.21) | 23.11 (22.47–23.74) |
| 2007 | 17.87 (17.31–18.43) | 29.38 (28.71–30.05) | 22.38 (21.91–22.85) | 24.35 (23.70–24.99) |
| 2008 | 17.63 (17.08–18.18) | 29.46 (28.80–30.13) | 21.81 (21.35–22.27) | 26.12 (25.46–26.78) |
| 2009 | 17.33 (16.79–17.88) | 27.93 (27.29–28.58) | 22.30 (21.84–22.76) | 25.74 (25.09–26.39) |
| 2010 | 17.10 (16.56–17.64) | 27.29 (26.65–27.92) | 21.87 (21.42–22.33) | 26.52 (25.86–27.17) |
| 2011 | 15.88 (15.37–16.40) | 26.85 (26.22–27.48) | 20.53 (20.10–20.96) | 26.53 (25.89–27.17) |
| 2012 | 16.45 (15.93–16.97) | 26.03 (25.42–26.64) | 20.01 (19.59–20.43) | 26.32 (25.69–26.95) |
| 2013 | 15.54 (15.04–16.04) | 25.42 (24.82–26.02) | 19.64 (19.23–20.05) | 28.71 (28.06–29.35) |
| 2014 | 15.04 (14.55–15.53) | 24.54 (23.96–25.13) | 18.87 (18.48–19.27) | 28.05 (27.42–28.68) |
| 2015 | 15.64 (15.14–16.13) | 25.96 (25.37–26.56) | 19.57 (19.17–19.97) | 30.40 (29.76–31.05) |
| 2016 | 16.66 (16.15–17.17) | 28.89 (28.27–29.52) | 22.09 (21.68–22.51) | 32.77 (32.10–33.43) |
| 2017 | 17.37 (16.85–17.88) | 30.95 (30.31–31.59) | 23.32 (22.90–23.75) | 35.59 (34.91–36.27) |
| 2018 | 18.09 (17.57–18.61) | 31.38 (30.74–32.02) | 24.12 (23.70–24.55) | 36.48 (35.81–37.16) |
| 2019 | 18.61 (18.09–19.14) | 31.49 (30.86–32.13) | 25.25 (24.82–25.67) | 36.67 (36.00–37.34) |
| 2020 | 24.86 (24.26–25.47) | 40.23 (39.52–40.94) | 31.89 (31.42–32.37) | 45.53 (44.79–46.27) |
| 2021 | 24.39 (23.80–25.00) | 43.30 (42.56–44.06) | 33.83 (33.34–34.34) | 49.69 (48.92–50.48) |
| 2022 | 22.83 (22.26–23.40) | 39.39 (38.69–40.09) | 32.27 (31.80–32.75) | 46.59 (45.86–47.33) |
| 2023 | 21.05 (20.51–21.60) | 37.98 (37.29–38.67) | 30.61 (30.15–31.07) | 43.32 (42.62–44.04) |
| 2024 | 21.47 (20.94–22.02) | 37.34 (36.67–38.02) | 30.20 (29.76–30.65) | 43.14 (42.45–43.84) |
| **Pooled 1999–2024** | **18.75 (18.64–18.85)** | **30.57 (30.44–30.70)** | **24.03 (23.94–24.12)** | **31.24 (31.11–31.38)** |

**Supplementary Figure 3.** Trends in T2DM and IHD-related age-adjusted mortality rates per 100,000, stratified by census region among adults aged 45 and above in the United States, 1999 to 2024.

Markers show observed rates; lines show Joinpoint-modeled trends (permutation test). APC = Annual Percent Change; AAPC = Average Annual Percent Change. *Significantly different from zero at α = 0.05.

**Supplemental Table 8.** State-level pooled T2DM and IHD-related age-adjusted mortality rates per 100,000 (95% CI) and their percentiles among adults aged 45 and above in the United States, 1999 to 2024.

| **State** | **Deaths** | **AAMR (95% CI)** | **Percentile** |
| --- | --- | --- | --- |
| Georgia | 9,051 | 11.01 (10.78–11.24) | 0 |
| Louisiana | 4,839 | 11.03 (10.72–11.34) | 2 |
| Massachusetts | 8,318 | 11.27 (11.03–11.52) | 4 |
| Nevada | 3,117 | 12.55 (12.10–13.00) | 6 |
| Connecticut | 5,176 | 12.65 (12.30–13.00) | 8 |
| New York | 30,633 | 14.63 (14.47–14.80) | 10 |
| District of Columbia | 840 | 15.23 (14.19–16.26) | 12 |
| New Jersey | 14,467 | 15.29 (15.04–15.54) | 14 |
| Mississippi | 5,028 | 17.40 (16.92–17.89) | 16 |
| South Carolina | 9,099 | 18.63 (18.25–19.02) | 18 |
| Alabama | 9,434 | 18.89 (18.51–19.28) | 20 |
| Utah | 3,762 | 19.02 (18.41–19.63) | 22 |
| Rhode Island | 2,410 | 19.15 (18.38–19.92) | 24 |
| Arizona | 13,241 | 19.20 (18.87–19.53) | 26 |
| Florida | 49,155 | 19.23 (19.06–19.40) | 28 |
| Arkansas | 6,091 | 19.40 (18.91–19.88) | 30 |
| Hawaii | 3,141 | 19.96 (19.25–20.66) | 32 |
| Illinois | 25,431 | 19.99 (19.74–20.24) | 34 |
| Alaska | 945 | 20.74 (19.34–22.14) | 36 |
| Virginia | 16,702 | 21.58 (21.25–21.91) | 38 |
| Delaware | 2,254 | 22.34 (21.41–23.27) | 40 |
| Maryland | 13,265 | 23.42 (23.02–23.83) | 42 |
| Colorado | 10,576 | 23.90 (23.43–24.36) | 44 |
| New Mexico | 5,084 | 24.50 (23.83–25.18) | 46 |
| Missouri | 16,093 | 24.94 (24.56–25.33) | 48 |
| New Hampshire | 3,641 | 25.17 (24.35–25.99) | 50 |
| Michigan | 27,525 | 25.92 (25.61–26.22) | 52 |
| Montana | 3,154 | 27.44 (26.47–28.40) | 54 |
| North Carolina | 27,211 | 28.27 (27.93–28.61) | 56 |
| Kansas | 8,540 | 28.44 (27.84–29.05) | 58 |
| Pennsylvania | 44,675 | 28.93 (28.67–29.20) | 60 |
| Texas | 61,244 | 29.54 (29.30–29.77) | 62 |
| Maine | 4,914 | 29.68 (28.84–30.51) | 64 |
| Idaho | 4,799 | 31.25 (30.36–32.14) | 66 |
| Indiana | 20,548 | 31.32 (30.89–31.75) | 68 |
| Nebraska | 6,269 | 31.65 (30.86–32.44) | 70 |
| Kentucky | 14,328 | 32.57 (32.04–33.11) | 72 |
| Wisconsin | 20,540 | 33.03 (32.57–33.48) | 74 |
| South Dakota | 3,218 | 33.87 (32.69–35.05) | 76 |
| Washington | 22,890 | 34.34 (33.89–34.79) | 78 |
| North Dakota | 2,806 | 35.19 (33.87–36.51) | 80 |
| Oregon | 14,996 | 35.39 (34.83–35.96) | 82 |
| Wyoming | 1,962 | 35.80 (34.20–37.40) | 84 |
| Minnesota | 20,083 | 36.08 (35.57–36.58) | 86 |
| Vermont | 2,637 | 36.65 (35.24–38.05) | 88 |
| California | 126,247 | 36.76 (36.55–36.96) | 90 |
| Tennessee | 25,463 | 39.13 (38.65–39.62) | 92 |
| Oklahoma | 15,435 | 40.31 (39.67–40.95) | 94 |
| Ohio | 51,107 | 40.44 (40.09–40.80) | 96 |
| Iowa | 15,127 | 41.85 (41.18–42.53) | 98 |
| West Virginia | 10,739 | 48.58 (47.66–49.51) | 100 |

Pooled by direct standardization across the 1999–2020 and 2018–2024 files. Three suppressed 2021–2024 cells (Delaware, District of Columbia, and Rhode Island, ages 45–54 years) were recovered from unsuppressed state totals.

**Supplemental Table 9.** CDC WONDER query parameters and saved query URLs.

Common parameters for all queries: Multiple Cause of Death files — 1999–2020 (bridged race; database D77) for 1999–2020 and 2018–2024 single race (database D157) restricted to 2021–2024; multiple cause of death = ICD-10 E11.0–E11.9 (non-insulin-dependent diabetes mellitus) AND I20–I25 (ischaemic heart diseases), listed anywhere on the death certificate (underlying or contributing); underlying cause of death unrestricted; ten-year age groups 45–54, 55–64, 65–74, 75–84, and 85+ years (age not stated excluded); all states and the District of Columbia; 2000 U.S. standard population; rates per 100,000. Pooled-rate queries used the same parameters with additional grouping by ten-year age group. Accessed October 19–21, 2026.

| **Output** | **Group by** | **Additional filters** | **URL (1999–2020 file)** | **URL (2021–2024 file)** |
| --- | --- | --- | --- | --- |
| Overall | Year | — | http://wonder.cdc.gov/controller/saved/D77/D525F336 | http://wonder.cdc.gov/controller/saved/D157/D525F379 |
| Sex | Sex; Year | — | http://wonder.cdc.gov/controller/saved/D77/D525F338 | http://wonder.cdc.gov/controller/saved/D157/D525F380 |
| NH race groups | Race (2021–2024: Single Race 6); Year | Hispanic Origin = Not Hispanic or Latino; 2021–2024: American Indian or Alaska Native, Black or African American, White | http://wonder.cdc.gov/controller/saved/D77/D525F356 | http://wonder.cdc.gov/controller/saved/D157/D525F381 |
| Hispanic | Hispanic Origin; Year | Hispanic Origin = Hispanic or Latino | http://wonder.cdc.gov/controller/saved/D77/D525F362 | http://wonder.cdc.gov/controller/saved/D157/D525F382 |
| NH Asian or Pacific Islander (2021–2024) | Year | Hispanic Origin = Not Hispanic or Latino; Single Race 6 = Asian + Native Hawaiian or Other Pacific Islander | — | http://wonder.cdc.gov/controller/saved/D157/D525F383 |
| Age-specific rates | Ten-Year Age Groups; Year | — | http://wonder.cdc.gov/controller/saved/D77/D525F371 | http://wonder.cdc.gov/controller/saved/D157/D525F386 |
| Census region | Census Region; Year | — | http://wonder.cdc.gov/controller/saved/D77/D525F372 | http://wonder.cdc.gov/controller/saved/D157/D525F389 |
| Metropolitan (1999–2020) | Year | 2013 Urbanization = Large Central, Large Fringe, Medium, Small Metro | http://wonder.cdc.gov/controller/saved/D77/D525F374 | — |
| Non-metropolitan (1999–2020) | Year | 2013 Urbanization = Micropolitan, NonCore | http://wonder.cdc.gov/controller/saved/D77/D525F375 | — |
| Urbanization deaths (2021–2024) | 2013 Urbanization; Year | Deaths only (population not released) | — | http://wonder.cdc.gov/controller/saved/D157/D525F604 |
| State | State | — | http://wonder.cdc.gov/controller/saved/D77/D525F376 | http://wonder.cdc.gov/controller/saved/D157/D525F388 |
| Place of death | Place of Death | — | http://wonder.cdc.gov/controller/saved/D77/D525F378 | http://wonder.cdc.gov/controller/saved/D157/D525F391 |

**Supplemental Table 10.** Joinpoint regression settings and permutation test results used to select the number of joinpoints for each series.

Settings (identical for all series): Joinpoint Regression Program version 5.2.0 (April 2024); grid search; log-linear model (ln y = xb); heteroscedastic errors using CDC WONDER standard errors, uncorrelated; minimum of 2 observations from a joinpoint to either end of the data and between joinpoints; minimum 0 and maximum 4 joinpoints; model selection by permutation test (4,499 permutations per test; overall significance level 0.05 with Bonferroni adjustment of each test); APC/AAPC confidence intervals by the empirical quantile method (5,001 resamples). No joinpoints were specified manually.

| **Series** | **Test** | **H0 vs H1 (joinpoints)** | **Permutation P value** | **Significance level** | **Joinpoints selected** |
| --- | --- | --- | --- | --- | --- |
| **Overall** |  |  |  |  |  |
| Overall | 1 | 0 vs 4 | <0.001 | 0.0125 |  |
|  | 2 | 1 vs 4 | <0.001 | 0.0167 |  |
|  | 3 | 2 vs 4 | <0.001 | 0.0250 |  |
|  | 4 | 3 vs 4 | 0.105 | 0.0500 | 3 |
| **Sex** |  |  |  |  |  |
| Female | 1 | 0 vs 4 | <0.001 | 0.0125 |  |
|  | 2 | 1 vs 4 | <0.001 | 0.0167 |  |
|  | 3 | 2 vs 4 | <0.001 | 0.0250 |  |
|  | 4 | 3 vs 4 | 0.078 | 0.0500 | 3 |
| Male | 1 | 0 vs 4 | <0.001 | 0.0125 |  |
|  | 2 | 1 vs 4 | <0.001 | 0.0167 |  |
|  | 3 | 2 vs 4 | <0.001 | 0.0250 |  |
|  | 4 | 3 vs 4 | 0.121 | 0.0500 | 3 |
| **Race and ethnicity** |  |  |  |  |  |
| NH American Indian or Alaska Native | 1 | 0 vs 4 | 0.003 | 0.0125 |  |
|  | 2 | 1 vs 4 | <0.001 | 0.0167 |  |
|  | 3 | 2 vs 4 | 0.037 | 0.0250 |  |
|  | 4 | 2 vs 3 | 0.019 | 0.0250 | 3 |
| NH Asian or Pacific Islander | 1 | 0 vs 4 | <0.001 | 0.0125 |  |
|  | 2 | 1 vs 4 | <0.001 | 0.0167 |  |
|  | 3 | 2 vs 4 | 0.961 | 0.0250 |  |
|  | 4 | 2 vs 3 | 0.996 | 0.0250 | 2 |
| NH Black or African American | 1 | 0 vs 4 | <0.001 | 0.0125 |  |
|  | 2 | 1 vs 4 | <0.001 | 0.0167 |  |
|  | 3 | 2 vs 4 | 0.002 | 0.0250 |  |
|  | 4 | 3 vs 4 | 0.242 | 0.0500 | 3 |
| NH White | 1 | 0 vs 4 | <0.001 | 0.0125 |  |
|  | 2 | 1 vs 4 | <0.001 | 0.0167 |  |
|  | 3 | 2 vs 4 | <0.001 | 0.0250 |  |
|  | 4 | 3 vs 4 | 0.065 | 0.0500 | 3 |
| Hispanic or Latino | 1 | 0 vs 4 | <0.001 | 0.0125 |  |
|  | 2 | 1 vs 4 | <0.001 | 0.0167 |  |
|  | 3 | 2 vs 4 | 0.352 | 0.0250 |  |
|  | 4 | 2 vs 3 | 0.406 | 0.0250 | 2 |
| **Age group (age-specific rates)** |  |  |  |  |  |
| 45–54 years | 1 | 0 vs 4 | <0.001 | 0.0125 |  |
|  | 2 | 1 vs 4 | <0.001 | 0.0167 |  |
|  | 3 | 2 vs 4 | 0.002 | 0.0250 |  |
|  | 4 | 3 vs 4 | 0.129 | 0.0500 | 3 |
| 55–64 years | 1 | 0 vs 4 | <0.001 | 0.0125 |  |
|  | 2 | 1 vs 4 | <0.001 | 0.0167 |  |
|  | 3 | 2 vs 4 | 0.005 | 0.0250 |  |
|  | 4 | 3 vs 4 | 0.102 | 0.0500 | 3 |
| 65–74 years | 1 | 0 vs 4 | <0.001 | 0.0125 |  |
|  | 2 | 1 vs 4 | <0.001 | 0.0167 |  |
|  | 3 | 2 vs 4 | 0.002 | 0.0250 |  |
|  | 4 | 3 vs 4 | 0.562 | 0.0500 | 3 |
| 75–84 years | 1 | 0 vs 4 | <0.001 | 0.0125 |  |
|  | 2 | 1 vs 4 | <0.001 | 0.0167 |  |
|  | 3 | 2 vs 4 | <0.001 | 0.0250 |  |
|  | 4 | 3 vs 4 | 0.127 | 0.0500 | 3 |
| 85+ years | 1 | 0 vs 4 | <0.001 | 0.0125 |  |
|  | 2 | 1 vs 4 | <0.001 | 0.0167 |  |
|  | 3 | 2 vs 4 | 0.002 | 0.0250 |  |
|  | 4 | 3 vs 4 | 0.103 | 0.0500 | 3 |
| **Urbanization (1999–2020)** |  |  |  |  |  |
| Metropolitan | 1 | 0 vs 4 | <0.001 | 0.0125 |  |
|  | 2 | 1 vs 4 | 0.008 | 0.0167 |  |
|  | 3 | 2 vs 4 | 0.298 | 0.0250 |  |
|  | 4 | 2 vs 3 | 0.148 | 0.0250 | 2 |
| Non-metropolitan | 1 | 0 vs 4 | <0.001 | 0.0125 |  |
|  | 2 | 1 vs 4 | <0.001 | 0.0167 |  |
|  | 3 | 2 vs 4 | 0.064 | 0.0250 |  |
|  | 4 | 2 vs 3 | 0.152 | 0.0250 | 2 |
| **Census region** |  |  |  |  |  |
| Northeast | 1 | 0 vs 4 | <0.001 | 0.0125 |  |
|  | 2 | 1 vs 4 | <0.001 | 0.0167 |  |
|  | 3 | 2 vs 4 | 0.006 | 0.0250 |  |
|  | 4 | 3 vs 4 | 0.252 | 0.0500 | 3 |
| Midwest | 1 | 0 vs 4 | <0.001 | 0.0125 |  |
|  | 2 | 1 vs 4 | <0.001 | 0.0167 |  |
|  | 3 | 2 vs 4 | <0.001 | 0.0250 |  |
|  | 4 | 3 vs 4 | 0.176 | 0.0500 | 3 |
| South | 1 | 0 vs 4 | <0.001 | 0.0125 |  |
|  | 2 | 1 vs 4 | <0.001 | 0.0167 |  |
|  | 3 | 2 vs 4 | <0.001 | 0.0250 |  |
|  | 4 | 3 vs 4 | 0.112 | 0.0500 | 3 |
| West | 1 | 0 vs 4 | <0.001 | 0.0125 |  |
|  | 2 | 1 vs 4 | <0.001 | 0.0167 |  |
|  | 3 | 2 vs 4 | 0.009 | 0.0250 |  |
|  | 4 | 3 vs 4 | 0.118 | 0.0500 | 3 |

At each step the null hypothesis (fewer joinpoints) is rejected when the permutation P value is below the Bonferroni-adjusted significance level; the final model is the last hypothesis retained.

**Supplemental Table 11.** Sensitivity analysis comparing models selected by the permutation test (primary) and the weighted Bayesian information criterion (WBIC).

| **Series** | **Permutation test: joinpoints, segment APCs (%)** | **WBIC: joinpoints, segment APCs (%)** | **Same model** |
| --- | --- | --- | --- |
| **Overall** |  |  |  |
| Overall | 3: 1999–2005: 3.51*; 2005–2015: -0.93*; 2015–2021: 9.06*; 2021–2024: -4.04* | 3: 1999–2005: 3.51*; 2005–2015: -0.93*; 2015–2021: 9.06*; 2021–2024: -4.04* | Yes |
| **Sex** |  |  |  |
| Female | 3: 1999–2005: 2.76*; 2005–2015: -2.06*; 2015–2021: 7.99*; 2021–2024: -4.38* | 3: 1999–2005: 2.76*; 2005–2015: -2.06*; 2015–2021: 7.99*; 2021–2024: -4.38* | Yes |
| Male | 3: 1999–2004: 4.92*; 2004–2015: -0.11; 2015–2021: 9.51*; 2021–2024: -3.84* | 3: 1999–2004: 4.92*; 2004–2015: -0.11; 2015–2021: 9.51*; 2021–2024: -3.84* | Yes |
| **Race and ethnicity** |  |  |  |
| NH American Indian or Alaska Native | 3: 1999–2003: 10.31*; 2003–2014: -0.95; 2014–2021: 7.12*; 2021–2024: -6.78* | 3: 1999–2003: 10.31*; 2003–2014: -0.95; 2014–2021: 7.12*; 2021–2024: -6.78* | Yes |
| NH Asian or Pacific Islander | 2: 1999–2014: 2.79*; 2014–2021: 9.77*; 2021–2024: -7.37* | 2: 1999–2014: 2.79*; 2014–2021: 9.77*; 2021–2024: -7.37* | Yes |
| NH Black or African American | 3: 1999–2006: 3.76*; 2006–2014: -2.21*; 2014–2021: 7.56*; 2021–2024: -4.26* | 3: 1999–2006: 3.76*; 2006–2014: -2.21*; 2014–2021: 7.56*; 2021–2024: -4.26* | Yes |
| NH White | 3: 1999–2005: 3.53*; 2005–2015: -1.19*; 2015–2021: 8.98*; 2021–2024: -3.05 | 4: 1999–2005: 3.64*; 2005–2014: -1.36; 2014–2018: 5.46; 2018–2021: 11.38*; 2021–2024: -3.92* | No |
| Hispanic or Latino | 2: 1999–2017: 1.60*; 2017–2020: 15.61*; 2020–2024: -4.76* | 2: 1999–2017: 1.60*; 2017–2020: 15.61*; 2020–2024: -4.76* | Yes |
| **Age group (age-specific rates)** |  |  |  |
| 45–54 years | 3: 1999–2003: 7.20*; 2003–2015: 1.36; 2015–2021: 11.87*; 2021–2024: -3.86 | 3: 1999–2003: 7.20*; 2003–2015: 1.36; 2015–2021: 11.87*; 2021–2024: -3.86 | Yes |
| 55–64 years | 3: 1999–2006: 2.83*; 2006–2014: -1.55*; 2014–2021: 9.58*; 2021–2024: -4.07* | 3: 1999–2006: 2.83*; 2006–2014: -1.55*; 2014–2021: 9.58*; 2021–2024: -4.07* | Yes |
| 65–74 years | 3: 1999–2005: 3.42*; 2005–2014: -1.76*; 2014–2021: 7.92*; 2021–2024: -5.55* | 3: 1999–2005: 3.42*; 2005–2014: -1.76*; 2014–2021: 7.92*; 2021–2024: -5.55* | Yes |
| 75–84 years | 3: 1999–2005: 3.64*; 2005–2015: -1.08*; 2015–2021: 8.38*; 2021–2024: -4.50* | 3: 1999–2005: 3.64*; 2005–2015: -1.08*; 2015–2021: 8.38*; 2021–2024: -4.50* | Yes |
| 85+ years | 3: 1999–2005: 3.75*; 2005–2015: -1.00*; 2015–2021: 8.99*; 2021–2024: -1.36 | 3: 1999–2005: 3.75*; 2005–2015: -1.00*; 2015–2021: 8.99*; 2021–2024: -1.36 | Yes |
| **Urbanization (1999–2020)** |  |  |  |
| Metropolitan | 2: 1999–2005: 3.83*; 2005–2014: -1.09; 2014–2020: 7.38* | 2: 1999–2005: 3.83*; 2005–2014: -1.09; 2014–2020: 7.38* | Yes |
| Non-metropolitan | 2: 1999–2005: 3.71*; 2005–2015: -1.73*; 2015–2020: 9.31* | 2: 1999–2005: 3.71*; 2005–2015: -1.73*; 2015–2020: 9.31* | Yes |
| **Census region** |  |  |  |
| Northeast | 3: 1999–2003: 3.36; 2003–2015: -2.43*; 2015–2021: 8.49*; 2021–2024: -5.03* | 3: 1999–2003: 3.36; 2003–2015: -2.43*; 2015–2021: 8.49*; 2021–2024: -5.03* | Yes |
| Midwest | 3: 1999–2005: 3.63*; 2005–2014: -2.50*; 2014–2021: 7.76*; 2021–2024: -3.59 | 3: 1999–2005: 3.63*; 2005–2014: -2.50*; 2014–2021: 7.76*; 2021–2024: -3.59 | Yes |
| South | 3: 1999–2005: 3.75*; 2005–2015: -2.02*; 2015–2021: 9.72*; 2021–2024: -3.51* | 3: 1999–2005: 3.75*; 2005–2015: -2.02*; 2015–2021: 9.72*; 2021–2024: -3.51* | Yes |
| West | 3: 1999–2008: 5.44*; 2008–2014: 1.22; 2014–2021: 8.05*; 2021–2024: -3.64* | 2: 1999–2018: 3.89*; 2018–2021: 11.80*; 2021–2024: -4.92* | No |

Identical models were selected for 17 of 19 series. *APC significantly different from zero (P < 0.05).
